# Extremely Low-Frequency Magnetic Field as a Stress Factor—Really Detrimental?—Insight into Literature from the Last Decade

**DOI:** 10.3390/brainsci11020174

**Published:** 2021-01-31

**Authors:** Angelika Klimek, Justyna Rogalska

**Affiliations:** Department of Animal Physiology and Neurobiology, Faculty of Biological and Veterinary Sciences, Nicolaus Copernicus University, 87-100 Torun, Poland; klimek@doktorant.umk.pl

**Keywords:** magnetic field, stress, HPA axis, catecholamines, cytokines, hormones, behavior, anxiety, neuroplasticity, cell survival

## Abstract

Biological effects of extremely low-frequency magnetic field (ELF-MF) and its consequences on human health have become the subject of important and recurrent public debate. ELF-MF evokes cell/organism responses that are characteristic to a general stress reaction, thus it can be regarded as a stress factor. Exposure to ELF-MF “turns on” different intracellular mechanisms into both directions: compensatory or deleterious ones. ELF-MF can provoke morphological and physiological changes in stress-related systems, mainly nervous, hormonal, and immunological ones. This review summarizes the ELF-MF-mediated changes at various levels of the organism organization. Special attention is placed on the review of literature from the last decade. Most studies on ELF-MF effects concentrate on its negative influence, e.g., impairment of behavior towards depressive and anxiety disorders; however, in the last decade there was an increase in the number of research studies showing stimulating impact of ELF-MF on neuroplasticity and neurorehabilitation. In the face of numerous studies on the ELF-MF action, it is necessary to systematize the knowledge for a better understanding of the phenomenon, in order to reduce the risk associated with the exposure to this factor and to recognize the possibility of using it as a therapeutic agent.

## 1. Introduction

**Figure 1:**
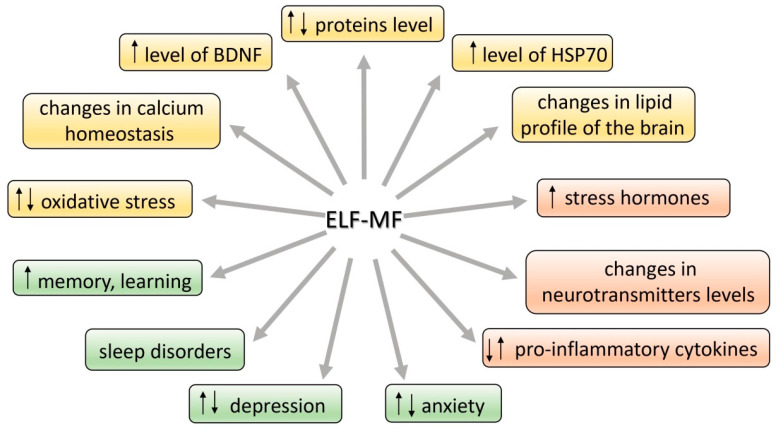
Effects of extremely low-frequency magnetic field (ELF-MF) action in the organism.

Many studies have suggested an association between extremely low-frequency magnetic field (ELF-MF) exposure and anxiety and/or depression. On the other hand, the ELF-MF-induced improvement of brain function has also been found. The mechanism of these effects is assumed to be a stress response induced by ELF-MF exposure. Extremely low-frequency MF is natural physical phenomenon in our environment. The rapid development of science and technology resulted in the introduction of many new devices and technologies in industry, agriculture, and everyday life. We are continuously exposed in our environment to ELF-MF (range of 0–300 Hz) [1]. MFs are either of natural origin (geomagnetic field, intense solar activity, thunderstorms) or human-made (factories, transmission lines, electric appliances at work and home, magnetic resonance imaging, medical treatment, etc.) [2]. Common used frequencies of electric and magnetic fields of the electric power supply and of electric and magnetic fields generated by electricity power lines and electric/electronic devices are 50 Hz in Europe and 60 Hz in North America [2]. Biological effects of ELF-MF and their consequences on human health have become the subject of important and recurrent public debate. Until now the reported studies are largely contradictory with regard to epidemiologic studies (some of the research studies found a relationship with development of diseases while the others failed to find any [3,4,5,6,7,8] (Table S1). Whether or not ELF-MF exposure is related to increased health risks, it has led many scientists to examine the potential mechanisms by which ELF-MF might affect human health. Special attention is paid to the adverse impact of both low- and high-frequency MF (radio waves) due to many possible pathological effects and numerous reports on MF-induced carcinogenicity [9]. ELF-MF was proved to be a stress factor and as a consequence, it can provoke morphological and physiological changes in stress-related systems [10]. Some authors argue that ELF-MF evokes cell/organism responses that are characteristic to general stress reaction. ELF-MF exposure “turns on” different intracellular—compensatory or deleterious—mechanisms and modifies stress-related function of nervous, hormonal and immunological systems (Figure 1). ELF-MF influence on living matter can cause a detrimental increase in free radicals levels and radical-evoked damages in macromolecules [11]. Most studies on ELF-MF effects concentrate on its negative influence; however, in the last decade there was an increase in the number of research studies showing stimulating impact of ELF-MF on brain plasticity processes (the production of protective proteins (e.g., Hsp70 or BDNF) or an increase in the activity of antioxidant enzymes) [12]. Furthermore, long-term exposure to ELF-MF can cause permanent changes in behavior (towards depressive and anxiety disorders) that are related to exposure to chronic stress [10,13,14].

In the face of numerous studies on the effects of the ELF-MF, it is necessary to systematize the knowledge for a better understanding of this phenomenon, in order to reduce the risk associated with exposure to this factor, but also to recognize the possibility of using it as a therapeutic agent.

## 2. Stress—A Factor Determining the Function of Organism at All Levels of Organization

It is accepted that ELF-MF exposure may count as a mild stress situation [10,15,16,17] and it could activate a wide spectrum of interacting neuronal, molecular, and neurochemical systems that underpin behavioral and physiological responses. Chronic stress can promote and exacerbate pathophysiology leading to allostatic overload in human body [18]. The brain developed some adaptive mechanisms in the face of changing environments and stress factors imposed on the nervous system. Integrated response to stressful stimuli is an essential component of adaptive processes critical for survival of the organism. Failure of this stress adaptation is considered as one of the primary neuropathological causes of stress-related disorders. A healthy organism is able to turn on or off effectively physiological and psychological responses to stimuli; however, if the stress system response is not adequate—too slow or too high, its mediators will enhance vulnerability to stress-related disease to which the individual is predisposed. Adaptation to repeated stress is associated with a complex cascade of molecular and cellular events, ranging from regulation of gene expression to release of neurotransmitters [19].

The definition of stress is not precise because the process is differently understood by people representing various fields of science. Stress can be discussed in the context of its influence on all levels of an organism’s organization: molecular, cellular, physiological, and behavioral as well as psychological. The term “stress” was introduced by Hans Selye [20] and described as a result of disturbed homeostasis in the organism. Seyle [21] stated that stress is a “nonspecific response of the body to any demand”. McEwen defined stress as “experiences that are challenging emotionally and physiologically” [18]. Other authors describe stress as a process involving perception, interpretation, response, and adaptation to harmful, threatening, or challenging events [22]. The reaction to a stress event is necessary for the organism to cope with danger [18]. Alarming signals include an internal, psychological, or environmental stimulus—such as ELF-MF. Some authors postulate that the changes turned on under the influence of exposure to ELF-MF are similar to those caused by other stress factors. The consequences of stress can be different and are dependent mainly on the strength of the stimulus. A low dose of stress can drive adaptive processes such as plasticity processes, e.g., the growth of postsynaptic (dendritic) spines, production of stress-resistant proteins, e.g., BDNF (brain-derived neurotrophic factor) and stimulation of neural stem cells to form new neurons that replace or cooperate with the existing ones [23], whereas even one high dose of a given factor may be harmful or even lethal [24].

In the neuroendocrine approach, stress is related to activation of the autonomic nervous system (SAM) and hypothalamo–pituitary–adrenal (HPA) axis. First, the autonomic nervous system is activated causing the release of noradrenaline and adrenaline from the adrenal medulla into the circulation, which—being a hormone—can rapidly regulate the function of peripheral organs [25] as well as the immunological response, which is supposed to adapt the organism to new, stressful conditions [26]. Acute activation of this system leads to release of noradrenaline from an extensive network of neurons throughout the brain, producing an enhanced state of arousal, which is critical for adaptive responses to stress [27]. Somewhat later, the HPA axis is activated, which causes the secretion of corticosteroid hormones from the adrenal cortex [25]. In response to a stressor, corticotropin-releasing hormone (CRH) is secreted in the hypothalamus. CRH is then driven with blood to anterior pituitary, where it causes adrenocorticotropic hormone (ACTH) release. In the next stage, ACTH reaches the adrenal glands and as a consequence glucocorticoids (cortisol and corticosterone) are secreted. The glucocorticoids cause increased arousal that ensures the organism’s readiness for action. Thus, the HPA axis system regulates the intensity, dynamics, and termination of the stress response [28,29]. Hippocampus, which role is the inhibition the HPA axis via the negative feedback, is of crucial importance for the dynamic of stress response [30,31,32]. On the other hand, corticosteroids can also modulate hippocampal function in the opposite directions: causing neuron’s dysfunction or plasticity and as a result, hippocampus-related behavioral changes can be observed. The hypothalamic–pituitary–adrenal (HPA) axis is sensitive to a broad spectrum of experimental and environmental events [30] that may result in physiological and behavioral changes in both directions: detrimental and compensatory ones. Sometimes these modifications are very subtle, but in some conditions they can even underlie the stress-related disorders or mediate the reversion of brain damage.

## 3. Molecular Stress Response to ELF-MF

Many studies show that stress induces the disruption in homeostasis [23,33] and as a consequence an overcompensation response is triggered to re-establish homeostasis. It needs gene expression and protein synthesis that progresses over time and leads to establish a new set-point for stress response systems. As ELF-MF is able to change the stress parameters, it is suggested that it can shift the set-point of endocrinological regulations and determine the health status of the organism as a consequence [15]. The effects of exposure to ELF-MF are particularly prevalent in the hippocampal area of the brain [34,35]. As mentioned, the hippocampus is involved in regulating the HPA axis activity, but on the other hand, stress hormones (mainly corticosterone and noradrenaline) are known to modulate hippocampal function and they may determine the plasticity processes in this area; it means an adaptive response to ELF-MF’s exposure. Targets for ELF-MF at molecular level include the cell membrane (e.g., its permeability, inorganic ion transport, receptor function), second messengers synthesis, chromosome structural changes and chemical changes in DNA structure, genes expression and protein synthesis (e.g., metabolism-related), free radicals, and neurotrophic factors. Such profound modifications have to be reflected in neurotransmitter activity, hormone release, and metabolism of the brain [36,37,38,39,40,41,42,43,44]. What is important, the effect of ELF-MF on molecular and/or cellular mechanisms is not obvious—it can be detrimental or protective. However, the research study on these mechanisms can shed some light on the possible metabolic pathway being possibly influenced by ELF-MF. ELF-MF-evoked cellular stress includes the modifications of key substances in cell metabolism—proteins and lipids. The mentioned alternations are mainly related to ELF-MF-induced oxidative stress. The consequence of these processes can be cell death such as apoptosis, necrosis, or autophagy [42,45,46,47,48].

### 3.1. Proteins and Lipids

As shown in in vivo research, ELF-MF can affect levels and function of proteins—crucial for maintenance of cell homeostasis, e.g., proteins anchored in lipid bilayer of the cell membrane functioning as ion channels, enzymes, and receptors, as well as the other proteins of key importance for the response to stress, regulation of apoptosis, and a number of metabolic processes [37,45,47,49,50]. Total protein level as well as its activity (e.g., alanine aminotransferase (ALT), aspartate aminotransferase (AST), alkaline phosphatase (ALP), albumin, bilirubin) was augmented in rats exposed to 1.5 mT ELF-MF [45]. Exposure to both 0.5 and 1 mT ELF-MF altered protein pattern in rat’s hippocampus. Gene ontology analysis showed that the most important function of the identified proteins altered after ELF-MF exposure is to ensure the functioning of the brain. Exposure to ELF-MF caused extreme downregulation of two proteins: Sptan1 and Dpysl2. The first is responsible for stabilization of cell membrane and organization of intracellular organelles. The second, Dpysl2, plays a key role in neuronal development and polarity, and additionally in neuron projection morphogenesis. Notably, the increased intensity of ELF-MF may be associated with more alteration in cell protein expression, and subsequent cell morphology and proliferation rate changes [47]. The chromogranin A (CgA) is another protein that should be mentioned as important in stress response and as a new target for electromagnetic radiation. It is a neuroendocrine secretory protein costored and coreleased with catecholamines from adrenal medulla, adrenergic nerve endings, and neuroendocrine cells. CgA is also a marker of sympathoadrenal activity, so its level gives information on the course of stress response [51]. The protein is also involved in maintaining calcium homeostasis in the cell [52]. What is important, its level increases during a depressive mood or stress situation [52]. The serum level of CgA in volunteer subjects chronically exposed to ELF-MF in the range 0.1–0.3 μT did not differ from the level in control group. However, a trend toward lower concentrations of CgA was observed in the group exposed to higher level of ELF-MF (>0.3 μT). Suppressive effects of ELF-MF on CgA level could be recognized as having inhibitory effects on the activity of the sympathetic nervous system [53].

In addition to proteins, the brain lipid profile is also influenced by ELF-MF exposure and taking into account multiple roles for lipids, they can be the medium for the ELF-MF action in the cell. Lipids are structural components of the cell membrane and they are involved in transfer of signals across membranes [17]. Apart from being structural elements, they are also required for axonal elongation and act as precursors for various secondary messengers, including arachidonic acid, docosahexaenoic acid, or 1,2-diacylglycerol [54]. Any changes in brain lipid metabolism lead to disturbances in homeostasis and are responsible for altered functioning at the cell and tissue levels. It was shown that 60 Hz 2.4 mT ELF-MF induces changes in the brain lipid profile and in corticosterone concentration. The level of these changes was similar to that in the positive control group of rats exposed to stress-RS (movement restraint). After 21 days of exposure to ELF-MF or RS or combined model (ELF-MF + RS), a general tendency to the decrease of total lipid level in brain structures was observed in each experimental group. Total cholesterol level was significantly increased in the cortex in the ELF-MF and RS  +  ELF-MF groups, and in subcortical structures in the RS + ELF-MF group. Inversely, polar lipids level in ELF-MF and RS + ELF-MF groups was decreased both in the cortex and in subcortical structures. Nonesterified fatty acid levels were found to be slightly higher in subcortical structures of the RS + ELF-MF group as compared to the control and RS groups. The analysis of fatty acid methyl esters revealed that the level of polyunsaturated fatty acids in cerebellum of ELF-MF-exposed rats was decreased, whereas their level in subcortical structures in the same group was increased. In addition to the changes in the amount of different kinds of lipids, the ELF-MF-induced lipid oxidative modifications were also noticed. The concentration of thiobarbituric acid reactive substances (TBARS, byproduct of lipid peroxidation) in lipids was higher, especially in the cortex and cerebellum of all treated groups [17]. Previous research has shown that immediate changes in lipid profile and TBARS levels after 2 h of singular exposure were visible only in the RS + ELF-MF group, whereas single exposure to ELF-MF or RS alone did not cause any changes in reduced glutathione and nitric oxide levels [55]. The increased level of lipid peroxidation was also noticed in rats exposed to ELF-MF (100 μT and 500 μT) [56]. The interesting research on ELF-MF-induced (50 Hz, 3 mT) changes in lipid profile (proteomic and transcriptomic profiling) in *Caenorhabditis elegans* was performed by Sun et al. [57]. In the glycerolipids (GLs) group, total triacylglycerols (TGs) content was increased while diacylglycerols (DGs) level was decreased. It should be also noted that among the most enriched proteins evaluated in this research, there were ones involved in lipid transport [57]. These studies indicate that ELF-MF affects the brain’s lipid balance in a similar way to physiological stressors.

### 3.2. Oxidative Stress and Antioxidant Status

Stress can be a factor causing an increase of the level of oxidative stress parameters in the brain, including lipid peroxidation and on the other hand, it can activate antioxidant response [58,59]. Oxidative stress is the result of an imbalance between reactive oxygen species (ROS) and antioxidants [29]. Under normal conditions the synthesis of ROS is usually balanced, but when the production of ROS increases they become harmful for organism. The imbalance causes changes at the cellular level, which causes DNA, proteins, and lipids damage. ROS are involved in physiological processes, for instance, in cell signaling and respiratory chain and immune response, but some pathological factors can contribute to their increased level [60]. Overproduction of ROS occurs, inter alia, in response to stress (heat, anoxia, ultraviolet light, injury, environmental pollution, cigarette smoke, psychological trauma, and many others) [61]. It has also been reported that ROS levels increase after ELF-MF exposure and the reason for this phenomenon can be the failure of antioxidant defense.

The disturbance of oxidative homeostasis was proved in in vitro research. Exposure to ELF-MF of 1 mT resulted in free radical increase in mouse macrophages [62] and SH-SY5Y neuroblastoma cells [39]. ELF-MF-induced increased ROS production was also found in K562 human leukemia cell line (50 Hz, 0.025/0.05/0.1 mT) [63,64], and in human osteoarthritic chondrocytes (100 Hz) [65]. The viability decrease and morphological changes of rat hippocampal neurons concomitantly with the increase of MDA (malondialdehyde) and ROS levels and reduction of superoxide dismutase activity were noticed after exposure to ELF-MF (50 Hz, 8 mT) [42]. Similarly, exposure to ELF-MF (50 Hz, 25–200 μT) resulted in increased ROS production and diminished activity of antioxidant enzymes (superoxide dismutase (SOD), glutathione peroxidase (GPx), glutathione reductase (GR)) in the human keratinocyte cell line NCTC 2544 [66].

In vitro results have been confirmed in in vivo research. The shift into oxidative processes, presented as ROS-level elevation and significantly, the total antioxidative capacity (TAC) level decrease, were found in *Caenorhabditis elegans* exposed to ELF-MF (50 Hz, 3 mT) [67]. These results, proving the ELF-MF-induced impairment of antioxidant mechanisms in the organism, were also obtained from research using rodent models. The toxic, increasing oxidative stress level effect of ELF-MF was found mainly in the brain. Akdag et al. [68] demonstrated that the activity of antioxidant enzyme catalase (CAT) was decreased in ELF-MF-exposed animals regardless of ELF-MF intensity (100 and 500 μT). Moreover, in the group exposed to 500 μT, TAC was lower than in the 100 μT group. At the same time, in the 500 μT group the levels of oxidative stress markers, MDA and MPO (myeloperoxidase), and values of total oxidant status (TOS) and oxidative stress index (OSI) were significantly higher. TBARS concentrations increasing concomitantly with decreasing reduced glutathione (GSH), total free-SH group concentrations, and TAC levels were found in rats exposed to ELF-MF (40 Hz, 7 mT and 50 Hz, 12 and 18 kV/m) [69]. The activities of antioxidant enzymes in brain homogenates were also decreased in rats exposed to ELF-MF (50 Hz 10 kV/m, 4.3 pT) [70]. In addition, in mouse brain subjected to ELF-MF (50 Hz, 8 mT), the levels of MDA, ROS, nitric oxide (NO), and nitric oxide synthase (NOS) were increased, whereas activities of SOD, CAT, and GPx were decreased [71,72]. Free radical level (superoxide anion- O_2_•^−^ and NO_2_^−^) was increased in the hypothalamus of rats exposed to ELF-MF (50 Hz, 10 mT) [73]. Acute exposure to ELF-MF (60 Hz 2.4 mT) resulted in the impairment of antioxidant mechanisms in the brain as well as in other tissues: heart, kidney, and plasma (decrease in SOD activity and reduced glutathione level) [55,74]. The disturbance of oxidative status was also found in testes of rats (diabetic model) exposed to ELF-MF (50 Hz, 8.2 mT): the increase in MDA and NO level, and diminished GSH level [75]. Many studies on the effects of ELF-MF have been conducted on people from risk groups, occupationally and residentially (living near high voltage lines) exposed to ELF-MF. El-Helaly and Abu-Hashem [76] carried out their research on a group of 50 electronic equipment installers and repairers. The serum malondialdehyde (MDA) level in the ELF-MF-exposed group was significantly higher than in control, and concomitantly the melatonin level (hormone supporting the antioxidant effect) in this group was lower. Similarly, the increment of oxidative stress and oxidative damage to DNA was also found in other research on power plant workers (occupational exposure, 110–420 kV and 4.09 V/m, 16.27 µT) [40,77,78]. The data suggest that exposure to ELF-MF could cause the failure of the antioxidant response and the collapse of homeostatic capability of the cell, leading to oxidative damage and functional impairment. However, the direct connection to the risk of disease development has not been unequivocally proved.

Subsequent studies shed light on the effects of ELF-MF on the antioxidant mechanisms that can underlie the protection against neurodegeneration. The ELF-MF-induced improvement of antioxidant protection has been evaluated in both in vitro and in vivo research. Exposure of C2C12 cells (myoblasts) to ELF-MF of 1 mT caused a drastic decrease in ROS level while total antioxidant status (TAS) and the activities of CAT and GPx were elevated [79]. Ehnert et al. [80] found ELF-MF-induced (16 Hz 6–282 μT) increase of SOD2, CAT, GPX3, and glutathione-disulfide reductase (GSR) activity concomitant with the reduction of ROS levels in human osteoblasts. Similarly, in the myelogenous leukemia cell line K562 exposed to ELF-MF (50 Hz, 1 mT) and in human blood platelets exposed to different sources of electromagnetic radiation (1 kHz, 0.5 mT; 50 Hz, 10 mT; or 1 kHz 220 V/m) CAT activity was increased [81,82]. Moreover, the exposure IMR-90 human lung fibroblasts for a total of 168 h to 6 mT ELF-MF contributed to decreased ROS level [83]. Exposure of human neuronal cell culture SH-SY5Y to 50 Hz ELF-MF with magnetic field intensity 1 mT resulted in elevated activity of NOS. This enzyme is controlled by proinflammatory cytokines that also activate ROS. After 1, 3, 6, and 24 h of exposure to ELF-MF, the activity of the enzyme was significantly increased. Moreover, the augmented production of O_2_^−^ was also found. However, CAT activity increased as the exposure time increased, possibly indicating a gradual adaptation of cells to the conditions of oxidative stress. On the other hand, these adaptive mechanisms turn out to be insufficient when ELF-MF exposure is combined with additional administration of H_2_O_2_—the oxidative effect is then exacerbated. These data suggest that ELF-MF may to some extent have neuroprotective effect. The combination of ELF-MF exposure and the stressor H_2_O_2_ prevents cells from being effectively defended against ROS [84]. However, when H_2_O_2_-treated cells were exposed to a higher value of ELF-MF (75 Hz, 2 mT), ROS level decreased and MnSOD activity increased [85]. It definitely suggests that the protective effect of ELF-MF depends on its intensity. The results of this in vitro research points out the beneficial effect of ELF-MF as an upregulation of antioxidant pathways, leading to protection against oxidative damage has been noted, reflecting an attempt to stimulate cellular response to neuronal damage.

In addition, ELF-MF as a mild stress factor activates an adaptive response that ensures the oxidative–antioxidant balance in rodent models as well as in humans. In a rat model of Huntington’s disease, ELF-MF (60 Hz and 0.7 mT) was found to be able to reverse the process of neuronal degeneration and oxidative stress; it enhanced the antioxidant glutathione content and reduced the oxidative stress markers, 8-hydroxy-2′-deoxyguanosine and oxidized glutathione levels, in the whole-brain tissue [12]. Recent research evaluating the redox state in post-stroke patients demonstrated the beneficial effect of ELF-MF on oxidative status. High magnetic intensity, 5 or 7 mT of 40 Hz ELF-MF, significantly increased enzymatic antioxidant activity as compared to results obtained before treatment. The results were correlated with the improvement in functional and mental status of post-stroke patients [86,87]. These data show that ELF-MF is a factor that may both increase the production of ROS and activate organisms’ antioxidant machinery in humans. In consequence, the electromagnetic radiation may drive the mechanisms underlying cell survival and plasticity.

### 3.3. Neuroprotective Proteins: Hsp70 and BDNF

Prosurvival responses include DNA repair processes and the increase in expression of chaperone protein—70-kDa heat shock proteins (Hsp70) and neurotrophin—brain-derived neurotrophic factor (BDNF) [88,89]. The expression of Hsp70 and BDNF appears to be a part of the general stress response and thus it is speculated to be associated with hormonal response to stress [89]. The increase of expression of these proteins would indicate the development of processes adapting neuronal networks in order to optimize circuits responding to the external environment and to integrate the response to challenges [90]. It was shown that stress hormones (mainly corticosterone and noradrenaline) influence via their receptors the plasticity processes in the hippocampus [31,91]. Noradrenaline can even dictate the direction of synaptic strength change in the hippocampus [91]. Under the influence of ELF-MF the expression of stress-response genes increases, resulting in higher levels of molecular chaperones such as Hsp70 [92,93,94]. The role of Hsp proteins is to stabilize polypeptide chains during their translocation across the cell membranes and to prevent aggregation of proteins with abnormal structure. Moreover, the antiapoptotic properties of Hsp70 and their role in appropriate folding and activation of proteins have also been proved [89,95]. As there are many pathways that could be affected to upregulate Hsp70 expression induced by stress, it is difficult to determine if any specific pathway may be affected.

The protective value of ELF-MF mediated by its influence on Hsp70 level was proved in in vitro research. Perez [96] showed that ELF-MF (50 MHz) leads to higher levels of Hsp70 in human T lymphocytes and fibroblast cell lines when subjected to stress, and that this response was of protective value. It seems to precondition and to enhance the cellular stress response when cells are provoked by toxic stimuli. Moreover, the cell protection was proportional to the levels of Hsp70. Exposure of human leukemia cell line K562 to ELF-MF (less than 0.1 and 1 mT) leads to increased Hsp70 levels [63,64]. More recent in vitro studies on ELF-MF with a density over 1 mT have shown marked effects, including an increase in Hsp70 transcription that results in protection against chronic hypoxia-induced injury [50].

Interesting data were also received in in vivo research on invertebrates. According to Gutzeit [97] exposure to 50 Hz ELF-MF with magnetic flux densities 50–150 μT enhances the response to thermal stress in *C. elegans*. ELF-MF-mediated specific genes activation could enhance transcription of an already activated set of heat shock genes by costressor (heat stress), thus providing an adequate and optimal defense response. Exposure to 60 Hz 8 µT ELF-MF caused regeneration of the heads and tails parts of the Planarian, *Dugesia dorotocethala*. This effect was accompanied by an increase in the level of Hsp70, which is triggered by extracellular signal-regulated kinase (ERK) cascade. It is known that ERK is activated as reaction to injury to promote regeneration [98].

In this approach, ELF-MF appears to be a mild stressor mobilizing the organism to cope with a dangerous situation [98]. The expression of the Hsp70 in response to stress serves to protect against the negative impact of stress. Hsp70 induction and stress systems function were shown to be two important inter-related mechanisms in maintaining the homeostasis under stress conditions [89]. According to the juxtaposition presented, some of beneficial effects of ELF-MF can be due to the protective role of Hsp70.

A substance of high importance for the nervous system is also brain-derived neurotrophic factor (BDNF). This neurotrophin is responsible for differentiation and survival of neurons during development, but it is also important for the adult brain, especially when subjected to stress conditions [90]. In a mature brain, BDNF ensures excitatory and inhibitory synaptic transmission and neuroplasticity [99]. The mechanism of neuroplasticity is crucial for learning and memory processes. It includes enhancement of the long-term potentiation (LTP), and stimulating and controlling neural growth. BDNF has a high affinity to full length tropomyosin receptor kinase B (TrkB) and its truncated isoform, p75 NTR. Through the activation of TrkB, the neurotrophin starts the cascade of signaling pathways, which results in neurogenesis, neuroplasticity, cell survival, and resistance to stress [100]. In vitro research proved that the exposure to pulsed ELF-MF (50 Hz; 1 mT for 2 h) increased the BDNF mRNA expression in cultured dorsal root ganglion neurons [101].

BDNF expression can be modulated by external, physiological, and pathological factors proven mainly in research on both rodents and humans. In the course of some diseases, such as Alzheimer disease, and during aging process or chronic stress, the inhibition of BDNF expression is noted, while exercise, enriched environment, and taking antidepressants are related to the intensified expression of BDNF [99]. ELF-MF is used in physical therapy due to its ability to stimulate BDNF synthesis. Several studies focused on this particular effect of ELF-MF on diseases and pathologies, like Huntington disease or stroke [12,102,103]. In post-stroke patients subjected to ten sessions of 15 min ELF-MF therapy (40 Hz 5 mT), plasma BDNF level was about 200% higher than before the treatment [102]. The study undertaken on a rat model of Huntington disease indicated that exposure to ELF-MF (60 Hz 0.7 mT 2 h in the morning and 2 h in the afternoon for 21 days) significantly elevated BDNF level in the rats with induced Huntington disease. Moreover, changes in the rats’ behavior related to Huntington disease were neutralized by ELF-MF [12]. Urnukhsaikhan et al. [103] showed that expression of BDNF, TrkB, and phosphorylated protein kinase B was increased in ELF-MF-stimulated (60 Hz, 10 mT) ischemic mice. In vitro research proved that the exposure to pulsed EMF (50 Hz; 1 mT for 2 h) increased the BDNF mRNA expression in cultured dorsal root ganglion neurons [103]. Thus, there is evidence suggesting that the neuroprotective effect of the exposure to extremely low-frequency MFs may be due to, at least in part, the impact of the fields on neurotrophic factors levels, leading to an increase of cell survival.

### 3.4. Plasticity, Neurogenesis, Proliferation, and Differentiation

The level of activity of voltage-gated Ca^2+^ channels is an important factor determining the synaptic transmission and leading to stimulation of short-term synaptic plasticity [104]. Calcium ions are involved in secretion of neurotransmitters. The influx of Ca^2+^ through presynaptic voltage-gated Ca^2+^ (Cav) channels triggers the release of neurotransmitters from presynaptic part of synapses. Measurements at a large glutamatergic synapse in the mammalian auditory brainstem—the calyx of Held—showed that vesicle endocytosis and synaptic transmission were enhanced in mice (8–10 postnatal days old) kept from birth under the influence of EMF (50 Hz EMF, 1 mT). Moreover, in mice exposed to EMF, the increase in expression of calcium channels at the presynaptic nerve terminal facilitating the influx of calcium was found. The observed mechanism is responsible for increasing endocytosis and synaptic plasticity [105]. In vitro research also evidenced the ELF-MF-induced increase in intracellular Ca^2+^ concentration. This effect was found in C2C12 cells (myoblasts) after 0.1 and 1 mT ELF-MF exposure [79], in human pluripotent stem cells (iPSCs) after 1.5 mT ELF-MF exposure [105], in dorsal root ganglion neurons after 0.1, 1, 10, and 100 mT ELF-MF application [101], and in rat hippocampal neurons exposed to 8 mT ELF-MF [42]. The influence of ELF-MF on proliferation and apoptosis and the participation of Ca^2+^ in these processes were also determined. In human neuroblastoma IMR32 and in rat pituitary GH3-cultured cells, the exposure to 1 mT 50 Hz ELF-MF caused the increased cell proliferation. At the same time, the increase of Ca^2+^ current density and of voltage-gated Ca^2+^ channel expression in the cell membrane was observed. In addition, blocking of Ca^2+^ channels by 15 μM Cd^2+^ alleviated the proliferative effect of ELF-MF. Apoptosis, induced by H_2_O_2_ or puromycin in IMR32 cells, was decreased after 72 h exposure to 1 mT 50 Hz ELF-MF. Blocking of L-type calcium channels by nifedipine also caused disappearance of the antiapoptotic effect of ELF-MF [106]. It has been also shown that ELF-MF influences calcium homeostasis in cultural entorhinal cortex neurons via calcium channel-independent mechanism. Twenty-four hour exposure to 1 or 3 mT ELF-MF does not affect voltage-gated calcium current and activity of calcium channels, but regulates intracellular calcium dynamics by decreasing the high-K^+^-evoked intracellular calcium elevation [107]. In summary, this study suggests that the change in calcium currents through voltage-gated calcium channels is the mechanism responsible for the proliferation promotion and antiapoptotic effect of ELF-MF.

Measurements at a large glutamatergic synapse in the mammalian auditory brainstem—the calyx of Held—showed that vesicle endocytosis and synaptic transmission was enhanced in mice (8–10 postnatal days old) kept from birth under the influence of ELF-MF (50 Hz ELF-MF,1 mT). Moreover, in mice exposed to ELF-MF, the increase in expression of calcium channels at the presynaptic nerve terminal facilitating the influx of calcium was found. The observed mechanism is responsible for increasing endocytosis and synaptic plasticity [108].

Throughout the life course, new neurons are continuously formed in the hippocampus, which is therefore a major site of structural plasticity in the adult brain. The existence of a causal link between ELF-MF-enhanced synaptic plasticity and neurogenesis has been shown by a number of in vivo experimental studies. ELF-MF (60 Hz, 0.7 mT, applied over 21 days) improved neurological scores, enhanced neurotrophic factor levels, and reduced neuronal loss in a rat model of Huntington’s disease [12]. In addition, prolonged exposure to ELF-MF (50 Hz, 100 µT; for 90 consecutive days; 2 h/day) increased LTP induction in rat’s hippocampus [35]. In vivo exposure of adult mice to ELF-MF (50 Hz, 1 mT) produced a marked increase in the number of newly generated neurons in the granule cell layer of the dentate gyrus [34]. Although the ELF-MF of 1 mT (for 21 days) caused the decrease in the dendritic spine density of neurons in hippocampus after 7 and 10 days, the effects disappeared after 14 days [109]. Studies on the rat traumatic brain injury model [110] and on rat Alzheimer’s disease model [49] have shown that ELF-MF reversed pathological brain damages and learning and memory abilities impairment. Similar effects were obtained after exposure of neurotoxin-injected mice to ELF-MF (50 Hz, 1 mT); the deficits such as neuronal maturation impairment, neurogenesis decrease, and memory disturbance decreased [111]. Studies on the beneficial effects of ELF-MF might yield fruitful insights related to clinical therapy of nervous-system-related diseases.

The cell differentiation at the expense of proliferation in different tissues and increased cell viability as an effect of exposure to low-frequency ELF-MFs is well evidenced in the literature. Collard et al. [112] reported an acceleration of proliferation and differentiation of human epidermis cells after exposure to low frequency (40 Hz) and demonstrated that the processes were related to a significant modification of gene expression. Falone et al. [85] found that ELF-MF (75 Hz, 2 mT) alone did not affect the viability of the human neuroblastoma SH-SY5Y cell line and that ELF-MF exposure prevented reduced cell viability after H_2_O_2_ application. Vannoni et al. [65] concluded that ELF-MF (100 Hz) stimulation is a useful tool to induce more divisions and thus to enhance cell proliferation of human osteoarthritic chondrocytes. The treatment of HeLa cells IMR-90 fibroblasts with ELF-MF (60 Hz, 6 mT) increased cell viability and activated cell cycle progression. In addition, ELF-MF mitigated the antiproliferative effect of GOx (agent stimulating H_2_O_2_ production) [83]. Di Loreto et al. [113] found that ELF-MF (50 Hz, 0.1–1 mT) had a positive effect on cell viability in primary cultures of maturing rat cortical neurons. The research of Ardeshirylajimi and Soleimani [105] on human pluripotent stem cells (iPSCs) suggested that ELF-MF (50 Hz, 1.5 mT) increases cell viability, division, proliferation, and mineralization of extracellular matrix. These results indicated that ELF-MF would improve the viability, proliferation, and differentiation of cells, and may be beneficial for the development of novel therapeutic approaches in regenerative medicine. The papers pointing to detrimental effect of ELF-MF on viability, differentiation, and proliferation should also be mentioned. It is, however, important that this effect is caused by high values of ELF-MF induction. Yin et al. [42] showed that the number of rat hippocampal neurons in G0/G1 phase was decreased and cells in S phase were accumulated as the effect of exposure to ELF-MF (50 Hz, 8 mT). The exposure of mesenchymal stem cells (bone marrow or adipose tissue derived) to ELF-MF of 20 mT (50 Hz) resulted in decreased cell proliferation [44,114]. This effect appeared to be related to the diminished expression of genes responsible for pluripotency and neuronal differentiation [44].

## 4. ELF-MF-Induced Changes in Levels of Neurotransmitters, Hormones, and Cytokines 

ELF-MF-induced molecular changes modify to a certain extent some crucial neuronal processes. As it is commonly known, the communication between main groups of signaling substances: neurotransmitters, cytokines, and hormones, is of high importance for the maintenance of health status of an individual. We have a lot of data confirming the effect of ELF-MF on functioning of nervous, immune, and endocrinological systems. However, the mechanisms by which the magnetic stimulation modulates the activity of these systems and the interplay between them are open to be identified. Up-to-date results concluded that the exposure of rats to ELF-MF may be sufficient to induce significant changes in the content of neurotransmitters. The levels of major inhibitory and excitatory amino acids and neurotransmitters: glutamate (Glu), glutamine (Gln), glycine (Gly), tyrosine (Tyr), and γ-aminobutyric acid (GABA), were elevated in the thalamus after five days of exposure to ELF-MF (60 Hz, 2 mT). In the striatum, higher levels of Gln, Gly and GABA were found as well, whereas their concentrations were decreased in cortex, cerebellum, and hippocampus. Dopamine level was increased in the thalamus [115]. Extremely low-frequency magnetic field (10 Hz; 1.8–3.8 mT) exposure was found to alter turnover and receptor reactivity of serotoninergic and dopaminergic systems and some behavioral disturbances induced by these systems [116]. The rats receiving chronic (10 days) repetitive transcranial magnetic stimulation (rTMS) treatment showed the symptoms of anxiety, and it was shown that the rTMS-induced anxiety might involve the serotonergic system [117]. The continuous exposure of rats to ELF-MF (50 Hz, 0.5 mT) affected cortical serotoninergic neurotransmission, and intensity of these changes depended on ELF-MF exposure duration [118]. The data may indicate the ability of ELF-MF to modify the function of main neurotransmitter systems and thus to modulation of some physiological processes, such as memory, emotionality, mood changes, sleep, alertness, or stress response. The response of individual brain tissues to exposure was varied; the level of one neurotransmitter increased in a given tissue appeared to be decreased in another, suggesting that the radiation can induce varying responses in the nervous system [115].

As noted, the existing data indicate that the exposure to ELF-MF may count as a mild stress situation and could be a factor in the development of disturbances of brain stress systems: hypothalamo–pituitary–adrenal (HPA) axis and sympatho–adrenal–medullary (SAM) system [10,16,115,119,120]. Although some findings indicate the deteriorating effects of magnetic fields on hormonal stress response, others failed to exhibit any obvious effects. Continuous long-term (4–6 week) ELF-MF (50 Hz, 0.5 mT) treatment induced some signs of stress: HPA-axis activation (elevated blood glucose level, elevated POMC (the precursor protein for ACTH) mRNA level, and enhanced depression-like behavior in a forced swimming test), although other markers of stress (elevated basal ACTH and corticosterone secretion, adrenal gland hypertrophy, thymus involution, loss of weight gain, and anxiety-like behavior in elevated plus maze) were not observed. This confirms that ELF-MF of the abovementioned intensity creates a weak stress response [10]. In addition, 50 Hz ELF-MF (0.207 μT) significantly raised ACTH, cortisol, and glucose levels in guinea pigs [15]. The concentration of plasma corticosterone level was significantly higher and remained at a similar level in groups of rats exposed to restraint stress (RS) or ELF-MF [17]. Research by Mahdavi et al. [121] showed that exposure to both 1 and 5 Hz ELF-MF of 0.1 mT intensity caused an elevation of ACTH level in rats’ plasma, whereas corticosterone level was reduced in both cases. In the animals exposed to 1 Hz ELF-MF, the concentration of adrenaline increased, but in rats exposed to 5 Hz, the level of adrenaline decreased. In rabbits exposed to ELF-MF (10 Hz ELF-MF), the level of blood corticosterone was increased in both the normal and high-cholesterol diet groups [122]. Chronic exposure (1 month) to 50 Hz 100/500 μT ELF-MF significantly raised corticosterone levels in rats’ plasma [123]. In mice exposed to 10 μT ELF-MF (1, 4, or 24 h/day for 1 week—short-term exposure), no significant differences in CRH gene expression in hypothalamus were observed, whereas ACTH plasma level was lower regardless of the daily exposure time (1, 4, or 24 h/day for 1 week). Moreover, the expression of pituitary level of POMC was lower in an exposure time-dependent manner, and a statistically significant decrease appeared after 24 h/day exposure [124]. Mostafa et al. [125] showed that 2- and 4-week exposure of rats to ELF-MF (2 G, equivalent to 0.2 mT) significantly increased their plasma corticosterone level. Other studies on mouse model showed that even a relatively low level of EMF (12 nT) can cause corticosterone increase [126]. On the other hand, Kitaoka [16] revealed that levels of ACTH, the hormone that regulates corticosterone secretion, and hypothalamic CRH and pituitary POMC were not changed by ELF-MF (70 Hz, 3 mT). Significant changes were also found in the levels of noradrenaline in various parts of rats’ brain: thalamus, hypothalamus, cerebellum and striatum, after 2 and 5 days exposure to 2 mT ELF-MF [115]. In the group of volunteers exposed to ELF-MF (50 Hz, 62 µT, for 2 h/day for 2 days with a 6-day interval) the cortisol level was increased at the beginning of ELF-MF exposure but later it diminished progressively [120]. The workers employed in the live-line procedures (132 kV high-voltage) for more than two years were found to be vulnerable for EM stress with altered adrenaline concentrations [40]. Moreover, exposure of turkey females to ELF-MF (50 Hz, 10 μT) caused NE-activated β-adrenoceptor function decrease, which is known to be involved in the formation of emotional disinterest and depression [126]. The data suggest that the exposure to ELF-MF can establish a new “set-point” for stress-system activity and the direction and dynamics of this process depend on the strength of the field and duration of exposure. The ELF-MF-induced changes in stress hormone levels will initiate cellular adaptation or damage by activation of intrinsic signaling pathways. Consequently, ELF-MF can change the vulnerability of the organism to subsequent stress factors and thus to diseases, mainly related to the nervous system.

Stress is known to strongly affect the immune system. It has been suggested that the potential contribution of ELF-MFs to anxiety or other stress associated disorders is also related to changes in the functioning of the immune system. Moreover, the chronic exposure to ELF-MF appears to also lead to immune system dysfunction, chronic allergic responses, inflammatory responses, and ill health [36]. However, as in other aspects of ELF-MF impact in organism, this factor can also be a double-edged sword and drive the survival-promoting processes. Importantly, the immune system and the stress systems—HPA axis and SAM—are closely linked to each other. Glucocorticoids and catecholamines are known to modify the secretion of cytokines: proteins that facilitate communication between the immune cells and the cells of the central nervous and endocrine systems [127]. Cytokines have the ability to modulate and activate the HPA axis. Proinflammatory cytokines: IL-1, IL-6, and TNFα, induce corticotropin-releasing hormone (CRH) secretion and they are also involved at every stage of stress reaction [128]. Changes in plasma proinflammatory cytokines were observed after acute continuous exposure (24 h) to ELF-MF with magnetic intensity of 7 mT. The levels of IL-1β, IL-6, and IL-2 were elevated. The number of white and red blood cells and lymphocytes, and the hemoglobin concentration and hematocrit level were increased. However, the repetitive exposure to ELF-MF (1 h/day for 7 days) did not alter either cytokines levels or blood parameters [129]. The change in cytokine production was also noticed in stroke patients treated with ELF-MF (40 Hz, 5 mT ELF-MF) [130]. Following the exposure to ELF-MF, the plasma levels of IL-1β and IL-2 cytokines and the level of IL-1β mRNA expression were increased. In addition, ELF-MF exposure increases the levels of the anti-inflammatory transforming growth factor β (TGF-β) and interleukin-18-binding protein [84]. Thus, the ELF-MF exposure can cause deregulation of the immune system, thereby increasing vulnerability to infectious and autoimmune diseases. Interesting results concerning the effect of ELF-MF on lymphocytes level were obtained by de Kleijn et al. [124]. In mice exposed to 10 μT ELF-MF (1, 4, or 24 h/day for 1 week—short-term exposure, or for 15-week long-term exposure) the increase in CD3+/CD4+ T-lymphocytes was observed only after short-term exposure to ELF-MF. The data suggest that the ELF-MF effect on immune and stress responses may be transient, because no changes in the number of immune cells were observed after long-term exposure. Several authors reported that the ELF-MF-evoked neuroplasticity can be mediated by the effect of magnetic radiation on cytokine level. Cytokines are found to influence the expression of neurotrophins and their receptors. This may indicate the role of inflammatory cytokines in the process of neuroplasticity [130]. The latest reports showed that 1 and 100 μT 50 Hz ELF-MF not only downregulates proinflammatory cytokines (IL-9 and TNF-α), but also activates an inflammation-suppressing cytokine, IL-10. In this case, the most noticeable effect was obtained at the highest value of magnetic induction [131]. The ELF-MF (60 Hz, 10 mT) also mitigated the deficits in ischemic mice, among others in the context of immune function: the levels of inflammatory mediators MMP9 and IL-1β were decreased [102]. The results demonstrate the recovery-stimulating potential of ELF-MF.

## 5. Association between ELF-MF Exposure and Emotional Behavior and Wellbeing

An association between ELF-MF exposure and emotional behavior has been indicated in many studies. The animal studies have shown that chronic exposure to ELF-MF may induce an anxiogenic and/or a depression-like effect. Dysfunction of stress systems can evoke negative emotional state and can potentiate fear- and anxiety-related behaviors [90,132]. Therefore, it is reasonable to speculate that elevation in ELF-MF-induced anxiety level may be attributed to the effect of ELF-MF on glucocorticoid release following activation of HPA axis and catecholaminergic sympathetic nervous system releasing adrenaline and noradrenaline. These pathways are key biological factors that modulate emotional behavior [90]. Liu et al. [133] reported that ELF-MF exposure (2 mT, 4 h/day for 25 days) had an anxiogenic effect in rats, such as anxiety-like behaviors in open field and elevated plus maze tests. Szemerszky et al. [10] demonstrated that ELF-MF exposure (0.5 mT, 4 weeks) in rats increased their immobility time in a forced swim test. The chronic exposure of mice to ELF-MF (3 mT, total exposure 200 h) induced the depression- and/or anxiety-like behavior (increase in total immobility time in a forced swim test and in the latency to enter the light box in a light–dark transition test). These behavioral disturbances were correlated with high corticosterone secretion [16]. Mice prenatally exposed to ELF-MF (50 Hz, 1 mT) lacked sociability and preference of social novelty, which can be a sign of autism-relevant social abnormalities; however, they did not show anxiety-like behavior [134]. The continuous (21 days) exposure to extremely low-frequency magnetic field (50 Hz, 10 mT) had no significant effect on activity and exploration activity but significantly increased stress and anxiety-related behavior in rats [135]. Quite similar observations in open field and elevated plus maze tests were described by Djordjevic et al. [73] after the exposure to ELF-MF (50 Hz, 10 mT) significantly reduced activity was observed. The noted effects of short-term ELF-MF exposure (50 Hz, 500 μT, 20 min) verified by behavioral tests in rats (elevated plus maze, novel object exploration) appear to suggest that these field parameters may cause some kind of discomfort, influence behavior, and increase passivity and situational anxiety [136]. Increased level of anxiety has also been found in rats exposed to ELF-MF of various flux density (50 Hz; 1, 100, 500, 2000 μT, and 2 mT) [56,137]. In accordance with this, Isogawa et al. [117] observed an anxiogenic effect of rTMS in rats tested in the elevated plus maze. It has also been shown that in rat pups after 6 weeks of exposure to ELF-MF (50 Hz, 3.5 mT, 1 h/day) behavior parameters, such as activity, motion, and response to sound and light, were decreased during exposure, but after exposure they settled back into normal control values [138]. However, the exposure of rats to ELF-MF of lower flux density (100 μT, 50 Hz, for 24 weeks) did not evoke any behavioral changes. The experimental group did not show any anxiety-like behaviors in open field or elevated plus maze. Similarly, depression-like behavior was not detected during tail suspension and forced swim tests [139]. Population studies paid attention to the role of ELF-MF in the development of sleep disorders, anxiety, and depression. It was shown that residential exposure to ELF-MF emitted by a radio–television broadcasting station could increase the anxiety in women [140]. Similarly, power plant workers (chronically exposed to ELF-MF) showed significantly poorer sleep quality than the unexposed group. Moreover, the level of depression symptoms in the exposed group was also significantly higher [14]. Interestingly, magnetic waves in the form of repetitive transcranial magnetic stimulation (rTMS) are used in therapy of depression. Three weeks of daily treatment caused a remission in a significant number of patients resistant to antidepressant treatment [13]. Similarly, 10-day treatment (20 × 2 s trains of 20 Hz stimulation with 58 s intervals) administered to patients with major depressive episodes significantly reduced scores in the Hamilton depression rating scale [141]. Exposure of post-stroke patients to 40 Hz, 7 mT ELF-MF 15 min/day for 4 weeks improved significantly cognitive functions and decreased up to 60% of depression syndromes [86]. As noted earlier, the possible explanation of beneficial effect of rTMS can be the ELF-MF-induced increase of mediators of corticosterone action in the hippocampus, i.e., neurotrophins, since these proteins appear to play a pivotal role in the structure and function of hippocampal neurons.

Behavioral effects of the ELF-MF depend on the length, frequency, and intensity of exposure, and on the initial balance of the brain transmitters [121,142]. Some authors noted reduced activity of animals after ELF-MF exposure, what is known as anxiety-like behavior. Others did not observe any changes following the exposure. Constant exposure to ELF-MF may also cause burdensome symptoms in humans, e.g., sleep disorders or depression. Notwithstanding, ELF-MF with high magnetic induction value (e.g., 7 mT—much higher than average exposure on a daily basis) appears to be effective in depression therapy. ELF-MF also improves cognitive functions in patients with a history of neurological injuries. However, again it should be noted that a considerable variety in the values of magnetic induction and exposure time were used in the research on ELF-MF effect.

## 6. Conclusions

Currently, people living in urbanized societies are exposed to the influence of various environmental stressors, including ELF-MF. The level of exposure can be different for individual groups and depends on the place of residence and occupation. The studies presented in this article indicate the possibility of changes at various levels of the organism organization as a result of exposure to ELF-MF. Effect of the field is observed in molecular and cellular responses, complex physiological processes such as activation of HPA axis and sympathetic system, as well as in behavioral and mood changes. As a result of exposure to ELF-MF, the homeostasis is disturbed in a way that is similar to the effect of application of any other stressor. The effects of ELF-MF are associated with the occurrence of various ailments, such as anxiety and sleep or mood disorders, but this type of stimulation is also successfully used in the therapy of depressive disorders as an alternative for drug-resistant or post-stroke patients. There is considerable evidence that ELF-MF-induced processes include interplay between the monoaminergic system, glucocorticoids, and neurotrophins [143]. Therefore, it is conceivable that changes in any of these elements of stress response may ultimately lead to changes in brain function and can be reflected in behavior. The ELF-MF-evoked initial disruption in homeostasis triggers an overcompensation response to re-establish homeostasis, which results in a bidirectional effects at the subsequent stages of response. Recent findings have elucidated the cellular signaling pathways and molecular mechanisms that mediate the character of response, which typically involve free radicals, antioxidants, protein chaperones (e.g., Hsp70) and growth factors (e.g., BDNF), hormones (mainly corticosterone and noradrenaline), cytokines, and neurotransmitters. Despite many studies on bioeffects of ELF-MF exposure, the picture is still not clear and unambiguous. However, we can try to make some general conclusions.

Summarizing the observations cited above, when the organism is subjected to the influence of ELF-MF, typical reactions for stimulation with a stress factor can be observed; however, the changes can evolve in both directions: detrimental or beneficial. It is possible to hypothesize that, as in the case of other stressors, while the exposure to milder ELF-MF (lower intensity and shorter duration) could promote neural plasticity, the chronic stressful conditions (high intensity and long-term duration) could sensitize limbic circuits resulting in greater susceptibility to damage. Some existing studies suggest that ELF-MF of low density creates the weak stress response and improves the brain function [10,15,121], but the effects of high density ELF-MF are definitely stronger as far as stress systems activation and behavioral impairment are concerned [16,119,135]. According to Directive 2013/35/EU and ICNIRP, 2010, the ELF-MF of flux density below 1 mT does not cause any changes in the organism, the field in the range of 1–6 mT may induce some temporary changes in the functioning of the nervous system, but the consequence of higher values of ELF-MF flux density can be permanent. However, other factors such as temporal features of exposure or individual hypersensitivity [144] are also important and can determine the consequences of this kind of stress on the organism. The review of literature concerning the ELF-MF impact on the organism showed that biological effects are often discussed in relation to intensity (T), frequency (Hz) of the field, and duration of exposure. However, the quantification of the electromagnetic phenomena in the organism—dosimetry—is of high importance for proper determining ELF-MF effects. Establishing reliable and reproducible measurement procedures is required. Thus, experimental studies should include the detailed characterization of internal electromagnetic fields in addition to other parameters of ELF-MF exposure. Improvement in the process of validation of physical aspects related to ELF-MF exposure is necessary to achieve the reliable answers to questions concerning the effects of ELF-MF on organism.

Still, there is no answer to the question of where the threshold for ELF-MF exposure lies, above which the adaptive possibilities of the organism are exceeded and when the direction of ELF-MF-induced processes turn into pathology. A better understanding of effects of ELF-MF at the cellular, molecular, physiological, and behavioral levels fills the gap in our knowledge of ELF-MF effects on stress response of systems activity. It is important to recognize the risk concerning the effects of magnetic flux density of ELF-MF on development of stress-related and neurodegenerative disorders. Understanding the fundamental mechanisms of these differential responses in neurons will lead to a new approach in risk assessment of ELF-MF exposure. On the other hand, the property of ELF-MF supporting rehabilitation will be successfully used in the development of novel approaches for the prevention and treatment of many different diseases.

## Supplementary Materials

The following are available online at https://www.mdpi.com/2076-3425/11/2/174/s1, Table S1: The influence of extremely low frequency magnetic field (ELF-MF) on different aspects of the organism function; http://doi.org/10.5281/zenodo.4457845.
