# Supplementary material for: Extremely Low-Frequency Magnetic Field as a Stress Factor—Really Detrimental?—Insight into Literature from the Last Decade
Source: Brain Sci. 2021 Jan 31;11(2):174. doi: 10.3390/brainsci11020174 (PMC7912337; doi:10.3390/brainsci11020174)
Supplement: Supplementary file 1 [file brainsci-11-00174-s001.pdf]

Table S1. The influence of extremely low frequency magnetic field (ELF-MF) on different aspects of the organism function (papers are listed according to year of publication).

In order to prepare the table the bibliography research in PubMed was performed using the following keywords in varied combinations:: “electromagnetic field”, “stress”, “corticosterone“, “noradrenaline”, “HPA axis”, “oxidative stress”, “cellular damage”, “BDNF”, “HSP70”, “neurotransmitters”, “cytokines”, “plasticity”, “viability”, “recovery”; “behavior”. We also used Boolean operator “and” to receive the most relevant search results. All articles written in English were manually screened, and the appropriate were identified. The articles published from 2010 to 2020 were taken into consideration. The papers concerning the effects of extremely low frequency magnetic field on a wide spectrum of molecular, neuronal, hormonal and behavioural stress responses were considered and only information related to ELF-MF effects are included in the Table.

	References/ Area of interest	Model	Parameters of exposure to ELF-MF	Procedure	Effects
1.	Akdag et al., 2010<br>**Oxidative stress**<br>**Cell death**	rat<br>(males, 4 months old,<br>n= 10)	50 Hz, 100 µT/500 µT	1.Rats were exposed to ELF-MF 2 h/day for 10 months.<br>2.Parameters assessed: after the last exposure<br>-active caspase-3 - apoptotic index<br>-MDA, TOS, OSI, MPO<br>-CAT, TAC	•active caspase-3 and MPO activity not changed<br>•CAT activity ↑<br>•OSI, TOS, MDA only after exposure to 500 µT ↑<br>•TAC was lower only after exposure to 500 µT ↓
2.	Cuccurazzu et al., 2010<br>**Calcium,**<br>**Proliferation**<br>**Proteins**<br>**Genes**<br>**Neuroplasticity**	C57BL/6 mouse<br>(males, adult, exposure<br>n= 38)	50 Hz, 1 mT	1.Mice were exposed (1) 7 h/day for 4 days, (2) 1 h/day, 3 h/day, 7 h/day for 7 days, (3) 7 h/day for 7 days.<br>2.Parameters assessed:<br>7h/day for 4 days:<br>-expression of pro-neuronal genes (*Mash1, NeuroD2, Hes1*); Expression of gene encoding Ca_v1- calcium channel subunits, Expression of proteins (NeuroD1, NeuroD2, Ca_v1 channels)<br>1 h/day, 3h /day, 7h /day for 7 days:<br>-Cell proliferation, Immune reactivity<br>7 h/day for 7 days:<br>-LTP	•gene expression of *Mash1, NeuroD2, Hes1* and gene encoding Ca_v1 calcium channels↑<br>•protein expression of NeuroD1, NeuroD2 and Ca_v1 ↑<br>•neurogenesis ↑<br>•newly generated immature neurons had survived and became mature dentate gyrus granule cells<br>•LTP ↑
3.	El-Helaly and Abu-Hashem, 2010<br>**Oxidative stress**<br>**Behaviour**	human<br>(mean age 36.88 years old,<br>n= 50)	50 Hz/60 Hz 0.06 µT-0.86 µT,	1.Workers chronically exposed to ELF-MF (mean employment duration 9-12 years)<br>2.Parameters assessed:<br>-sleep sufficiency<br>-plasma melatonin level<br>-MDA level	•melatonin ↓<br>•MDA ↑<br>•sleep insufficiency ↑

4.	Frahm et al., 2010<br>**Oxidative stress**<br>**Proteins**	mouse macrophage s<br>from bone marrow	50 Hz, 1 mT	1.Cells were exposed to ELF-MF from 5 min to 24 h.<br>2.Parameters assessed:<br>-reactive oxygen species after 5 , 15, 30 and 45 min of ELF-MF exposure.<br>-regulatory proteins and proteins involved in the response to oxidative stress levels (gp91phox, clathrin and adaptin) after 15, 30, 45 min and 1, 2, 4 or 24 h of exposure<br>-the levels of PI3K, PKB and PP2A after 5, 10, 15, 30, 45 min and 1 or 24 h of exposure.	•ROS production ↑<br>•clathrin, adaptin, PI3K, PKB, PP2A ↓<br>•not changed or slightly increased level of gp91phox after short term exposures (< 2h)
5.	Garip and Akan, 2010<br>**Oxidative stress**<br>**Cell death**<br>**Viability**<br>**Proteins**	K562 human leukemia cell line	50 Hz, 1 mT	1.Experimental groups:<br>-cells treated with H_2O_2 to induce apoptosis<br>-cells exposed to ELF-MF for 3 h<br>-cells H_2O_2 treated and exposed to ELF-MF for 3 h<br>2. Parameters assessed:<br>- number of apoptotic cells,<br>- ROS and Hsp70	•ELF-MF exposure alone ↓ in number of apoptotic cells.<br>•in H_2O_2 treated cells ELF-MF significantly enhanced apoptosis level<br>•viability of ELF-MF exposed cells not changed<br>•H2O2-induced decreased cells viability not altered by ELF-MF.<br>•ROS level ↑ in all ELF-MF exposed groups<br>•level of Hsp70 ↑ in all ELF-MF exposed groups
6.	George et al., 2010<br>**Behaviour**	depressive patients (mean age 47,7 years old, n= 92)	10 Hz, 120% of motor threshold 3000 stimuli per session	1.Patients subjected to 3-week treatment with rTMS (40 min/day in a 5-day sequence- 15 sessions).<br>2.Parameters assessed:<br>-Hamilton Scale for Depression score	•Hamilton Scale for Depression score ↑<br>•the odds of attaining remission ↑
7.	Goraca et al., 2010<br>**Oxidative stress**	rat<br>(males, 2-3 months old, n= 7)	40 Hz, 7 mT	1. Rats were exposed to ELF-MF 30 min/day or 60 min/day for 14 days.<br>2. Parameters assessed (after the last exposure)<br>- TAC level in plasma<br>- TBARS, H_2O_2, total free sulphydryl groups and GSH concentrations in heart tissue.	•ELF-MF exposure 30 min/day – no effect<br>•ELF-MF exposure 60 min/day:<br>-TBARS and H_2O_2 concentrations ↑<br>-GSH, total free -SH groups, TAC level ↓
8.	Mannerling et al., 2010<br>**Oxidative stress**<br>**Proliferation**<br>**Viability**<br>**Proteins**	K562 human leukaemia cell line	50 Hz, 0.025 mT/0.05 mT/ 0.1 mT	1.Cells were exposed to ELF-MF of 0.1 mT for 1 h or to heat shock (42ºC) (positive control), then incubated 24 h.<br>Parameters assessed:<br>proliferation, viability and cell cycle distribution<br>2.Cells were exposed to ELF-MF of several flux densities	•0.1 mT ELF-MF no effect on proliferation or cell cycle<br>•Hsp70 expression after ELF-MF at several flux densities ↑<br>•free radical release ↑ at each flux density

				Parameter assessed:<br>-Hsp70 level and superoxide anion radical immediately, 6, 12 and 24 h after exposure.	
9.	Martínez-Sámano et al., 2010<br>**Oxidative stress**	rat<br>(males, adult, n= 6)	60 Hz, 2.4 mT	1.Rats (restrained and unrestrained) were exposed to ELF-MF for 2 h.<br>2.Parameters assessed:<br>-GSH, CAT, SOD and TBARS in liver, heart, kidney and plasma immediately after exposure	•CAT activity and TBARS levels not changed in all groups<br>•SOD activity ↓ in plasma unrestrained ELF-MF exposed group and unchanged in the restrained group exposed to ELF-MF<br>•GSH concentration ↓in unrestrained exposed group in heart, kidney and plasma and in restrained group exposed to ELF-MF in heart and liver
10.	Morabito et al, 2010<br>**Oxidative stress**<br>**Calcium**	C2C12 cells (myoblasts)	50 Hz, 0,1 mT/1 mT	1.Cells were exposed to ELF-MF for 30 min.<br>2.Parameters assessed:<br>-reactive oxygen species (intracellular superoxide anion, hydrogen peroxide)<br>-TAS, activity of antioxidant enzymes<br>-cell damage markers (protein carbonyl content, MDA);<br>-mitochondrial membrane potential<br>-Ca^{2+} signaling	•0.1 mT ELF-MF no effect on ROS production<br>•1 mT:<br>- superoxide anion level not changed; hydrogen peroxide and TAS ↑<br>-MDA and protein carbonyl content not changed<br>-activity of GPx ↑<br>-activity of CAT ↑<br>•basal intracellular Ca^{2+} after 0.1 and 1 mT exposure ↑
11.	Szemerszky et al., 2010<br>**Behaviour**<br>**Stress hormones**	rat<br>(males, adult, n= 8)	50 Hz, 0.5 mT	1.Rats were subjected into short term (8 h/day for 5 days) exposure to ELF-MF.<br>Parameters assessed:<br>-elevated plus maze (48 h after exposure)<br>-ACTH, POMC and CORT (48 h after exposure and 2 days later)<br>2.Rats were subjected into long term (24 h/day for 6 weeks) exposure to ELF-MF.<br>Parameters assessed:<br>-forced swim test (in the 4th week)<br>-week elevated plus maze (in the 6th test)<br>-ACTH, POMC and CORT (in the 6th test)	•elevated plus maze behaviour – not changed<br>•helpless behaviour ↑<br>•POMC ↑ (long term exposure)<br>•ACTH and CORT levels – not changed
12.	Emre et al., 2011<br>**Oxidative stress**<br>**Cell death**	rat<br>(males, adult, n= 10)	1 Hz/10 Hz/ 20 Hz/40 Hz, 1.5 mT pulsed ELF-MF	1.Rats were exposed to ELF-MF of frequencies 1 Hz, 10 Hz, 20 Hz and 40 Hz in sequence with 4-min and 1-min intervals between each frequency 1 h/days for 30 days.<br>2.Parameters assessed (after the last exposure):	•ALT, AST and ALP activities as well as albumin, bilirubin and total protein levels ↑<br>•MDA concentration and SOD activity were increased ↑<br>•necrotic cell ↓

	**Proteins**			-ALT, AST,ALP activities, albumin, bilirubin and total protein levels in serum.<br>-MDA concentration and SOD activity in liver<br>-apoptotic and necrotic cells	•apoptotic cells ↑
13.	He et al., 2011<br>**Behaviour**	rat<br>(males, adult, n= 10)	50 Hz, 2 mT	1.Rats were exposed to ELF-MF for 1 h or 4 h per day for 4 weeks.<br>2. Parameters assessed :<br>- behaviour in open field test, elevated plus maze and Morris water maze after last exposure	4-weeks exposure to ELF-MF for 4 h/day:<br>•anxiety- like behaviours ↑<br>•latency to find hidden platform in Morris water maze ↓<br>•long-term memory of former location of platform ↑<br>•short-term memory and locomotor activity – not changed
14.	Hosseini et al., 2011<br>**Oxidative stress Stress hormones**	rabbit<br>(males, adult, n= 8)	10 Hz pulsed ELF-MF	1.Rabbits with normal and high-cholesterol diet were exposed to ELF-MF 2 h/day for 5 days.<br>2.Parameters assessed (12 h after the last exposure)<br>-plasma levels of CORT, free-T3, free-T4 and MDA	•CORT, free-T3 and free-T4 in exposed rabbits with normal and high-cholesterol diet ↑<br>•MDA in exposed rabbits with high-cholesterol diet ↓
15.	Juszczak et al., 2012<br>**Cell death**	rat urothelial cultured cells	50 Hz, 45 mT pulsed ELF-MF	1.Urothelial cells were exposed to pulsating ELF-MF three times for 4 h with 24-h intervals.<br>2. Parameters assessed:<br>-apoptotic and necrotic cells after exposure	•apoptosis ↑<br>•necrosis ↓
16.	Kirschenlohr et al., 2012<br>**Cell death Proliferation Stress hormones Genes**	human<br>(males, 20-30 years old, n= 17)	50 Hz, 62 μT	1.Group of volunteers was exposed to ELF-MF for 2 h/ day for 2 days with 6 days interval.<br>2.Parameters assessed:<br>-expression of genes related to stress response, cell proliferation, apoptotic genes and CORT concentration in plasma at the time points: 0 min, 5 min, 10 min, 20 min, 40 min, 80 min and 120 min of experiment	•no gene response<br>•CORT level ↑ the beginning of ELF-MF exposure but diminished progressively
17.	Kitaoka et al., 2012<br>**Behaviour Stress hormones Genes**	mouse<br>(males, 4 weeks old, n= 5-10)	60 Hz, 3 mT	1.Mice were exposed to ELF-MF 8 h/day for 25 days.<br>2.Parameters assessed:<br>-behaviour in open field test, elevated plus maze, light–dark transition test and forced swim test (after exposure)<br>-noradrenaline, ACTH and CORT, glucose, the expression of genes related to stress response (week after behavioural tests)	•anxiety- like behaviours ↑<br>•helpless behaviour ↑<br>•ACTH – unchanged<br>•CORT ↑<br>•glucose - unchanged<br>•CYP17A1 expression ↑<br>•noradrenaline release and adrenal tyrosine hydroxylase expression - unchanged

18.	Korpinar et al., 2012 **Behaviour**	rat (males, adult, n= 38)	50 Hz, 10 mT	1.Rats were exposed to ELF-MF 24h/day for 21 days.<br>2.Parameters assessed (after exposure) :<br>-behaviour in elevated plus-maze and hole-board tests	•stress and anxiety- like behaviours ↑<br>•activity and exploration – not affected
19.	Martínez-Sámano et al., 2012 **Oxidative stress Stress hormones Lipids**	rat (males, 45 days old, n= 8)	60 Hz, 2.4 mT	1.Rats (restrained (ELF-MF+RS) and unrestrained (ELF-MF)) were exposed to ELF-MF for 2 h.<br>2.Parameters assessed (after exposure)<br>-SOD and CAT activities, reduced GSH, NO,<br>-total cholesterol, and triacylglycerol levels, TBARS content in total lipids in brains<br>-plasma CORT concentrations.	•SOD and CAT activities ↓ in ELF-MF and ELF-MF+MR exposed rats<br>•GSH and NO levels ↓ in ELF-MF+RS treated group<br>•total cholesterol and triacylglycerol levels not affected<br>•TBARS in total lipids ↑ in ELF-MF+RS group<br>•CORT level not changed in ELF-MF group
20.	Tasset et al., 2012 **Oxidative stress Neuroplasticity Behaviour Neurotransmitters**	rat (males, 3 months old, n= 8)	60 Hz, 0.7 mT	1.Rats (3-Nitropropionic acid administrated -3NP to induce Huntington disease) were exposed to ELF-MF 2 h in the morning and 2 h in the afternoon for 21 days.<br>2.Parameters assessed (after exposure)<br>-behavioural changes in open field and forced swim tests<br>-BDNF, GDNF and dopamine levels<br>-production od lipid peroxidation products 8-hydroxy-2'-deoxyguanosine (8-OHdG) and GSH as well as capsase-3 and LDH activities	ELF-MF effects in 3NP treated rats:<br>•neutralization of behavioural disturbances<br>•recovery of dopamine levels<br>•BDNF, GDNF ↑<br>•cell damage and oxidative stress markers ↓<br>•reversed neurodegeneration
21.	Vannoni et al., 2012 **Oxidative stress Proliferation Viability**	human osteoarthritic chondrocytes	100 Hz ELF-MF or Musically Modulated Electromagnetic Fields	1.Cultured cells were exposed 30 min/day for 15 days to ELF-MF or a system of Therapeutic Application of Musically Modulated Electromagnetic Fields (TAMMEF) of variable frequency and intensity.<br>Parameters assessed:<br>-cell survival and proliferation, expression of ERK1/2 proteins 3, 7 and 15 days of exposure<br>-ROS and reduced GSH production during 15 days of exposure.<br>-Mitochondrial transmembrane potential<br>2.Cells were stimulated with IGF-1 or IL-1β (model of osteoarthritis) and treated for 12 h ELF-MF or TAMMEF.<br>Parameters assessed:	•cell proliferation ↑ both types of field<br>•expression of ERK1/2 proteins ↑ in ELF-MF exposed cells<br>•apoptosis level and mitochondrial transmembrane potential unchanged by both types of field<br>•at the beginning of the ELF-MF treatment, the levels of ROS and reduced GSH ↑, but returned to lower levels during consecutive days of exposure<br>•ROS production not altered by ELF-MF in IGF-1 and IL-1β stimulated cells

				-ROS and reduced GSH production,<br>-mitochondrial transmembrane potential	•restored mitochondrial transmembrane potential and GSH ↑ after both types of exposure in IGF-1 and IL-1β induced cells
22.	Yang et al., 2012<br>**Cell death**<br>**Proteins**<br>**Behaviour**	rat<br>(males, n= 32)	15 Hz,<br>0.1 mT/0.3 mT/0.5 mT	1. Rats (traumatic brain injury model) were exposed ELF-MF for 30 min, 1 h, 6 h, 12 h, 18 h, 24 h or 30 h. From 30 min to 30 h after ELF-MF exposure animals were injected with kainic acid to induce apoptosis.<br>2. Parameters assessed:<br>-Morris water maze - 24 h after kainic acid injection<br>-apoptosis level, brain water content and blood-brain barrier damages<br>-HIF-1 protein expression	•memory ↑<br>•apoptosis ↓<br>•brain water content ↓<br>•blood-brain barrier damages ↓<br>•HIF-1 protein expression ↓
23.	Amaroli et al., 2013<br>**Oxidative stress**<br>**Viability**<br>**Proteins**	protozoan *Dictyostelium discoideum*	50 Hz 300 μT	*1.D. discoideum* cells were exposed to ELF-MF for 24 h.<br>*2.*Parameters assessed:<br>-cell growth, pseudocholinesterase activity and Hsp70-related molecules and<br>-CAT and GPx activities immediately after exposure and 24 h later	•cell growth ↓<br>•activity of pseudocholinesterase ↑<br>•Hsp70-related molecules ↑<br>•CAT and GPx activities unchanged<br>•effect transient - all altered parameters returned to their control values 24-h after ELF-MF exposure
24.	Collard et al., 2013<br>**Cell death**<br>**Proliferation**<br>**Genes**	human epidermis cultures	40 Hz pulsed ELF-MF	1.Epidermis cultures were exposed to pulsed ELF-MF 40 min/day for 11 days.<br>2.Parameters assessed:<br>expression of genes involved in proliferation, differentiation, apoptosis, cell migration and stress response 4 and 7 days of exposure and at the day after the end of the exposure	•expression of genes involved in proliferation, differentiation, apoptosis, cell migration and stress response (*DKK1, SPRR3, NDRG4, CHEK1*) ↑
25.	Corallo et al., 2013<br>**Oxidative stress**<br>**Proteins**<br>**Immune response**<br>**Viability**	human osteoarthritic chondrocytes	100 Hz ELF-MF<br>or<br>Musically Modulated Electromagnetic Fields	1.Cultured cells were exposed 30 min/day for 14 days to ELF-MF (100 Hz) or a system of Therapeutic Application of Musically Modulated Electromagnetic Fields (TAMMEF).<br>2.Parameters assessed:<br>-cell viability at days 2, 7 and 14<br>-proteomic analysis	•cell viability ↓ in ELF-MF exposed cells<br>•protein involved in inflammatory response: S100-A10 ↑ after TAMMEF exposure and S100-A11 ↑ after ELF-MF exposure<br>•cystatin-B proteinase inhibitor ↑ both types of field<br>•MnSOD ↓ after ELF-MF exposure and ↑ after TAMMEF<br>•pattern of proteins associated with cell metabolism - changed by both types of field
26.	Duan et al., 2013	mouse	50 Hz, 8 mT	1.Mice were exposed to ELF-MF (4 h/day for 28 days).	•learning and memory abilities ↓<br>•MDA, ROS, NO and NOS ↑

	**Oxidative stress Behaviour**	(males, 3 weeks old, n= 10)		2.Parameters assessed:<br>-learning and memory (Morris water maze) after exposure then<br>-ROS, MDA and NO levels as well as NOS, SOD, CAT and GPx activities	•activities of SOD, CAT and GPx ↓
27.	Li et al., 2013 **Genes**	*Drosophila melanogaster* (males, after eclosion or from egg stage, n= 20- pre-screening, 60- further analysis)	50 Hz, 3 mT	1.Insects were treated with short- term (8 h/16 h/24 h/48 h/ 72 h) and long- term (lifetime) ELF-MF exposure.<br>2.Parameters assessed: in three-day old flies<br>-transcriptomic analysis of genes	•short-term exposure - affected genes involved in metabolic processes, cytoskeletal organization, mitotic spindle organization, cell death, protein modification and proteolysis.<br>•long-term exposure - changed expression of genes involved in metabolic processes, response to stress, mitotic spindle organization, aging, cell death and cellular respiration.
28.	Alsaeed et al., 2014 **Behaviour**	mouse (males, n= 8)	50 Hz, 1 mT	1.Pregnant females were exposed to ELF-MF in the last week of gestation, their offspring was then exposed to the same field conditions 7 days after birth.<br>2.Parameters assessed (8-11 weeks of age):<br>- sociability (Crawley's test, preference for social novelty test<br>- behaviour in open field test, elevated plus maze, hole-board<br>- olfactory abilities and motor coordination	•sociability and preference of social novelty – lacked<br>•locomotion – unchanged<br>•anxiety-like behaviour – not showed<br>•exploratory behaviour ↓<br>•olfactory abilities and motor coordination - unchanged
29.	Giorgi et al., 2014 **DNA damage**	human neuroblastoma BE(2)C cells	50 Hz, 1 mT pulsed ELF-MF	1.Cell were pre-treated with H_2O_2 (300 μM, 1 h) and then incubated under 1 mT pulsed ELF-MF 1, 24, 48 and 72 h after H_2O_2 treatment.<br>2.Parameters assessed:<br>-DNA damage<br>-cytotoxicity.	•H_2O_2- - induced DNA damages – not influenced<br>•cytotoxic effect of H_2O_2.- not affected
30.	de Groot et al., 2014 **Oxidative stress Calcium**	pheochromocytoma (PC12) cells	50 Hz, 1 μT-1000 μT block-pulsed ELF-MF	1.Chemically stressed or untreated PC12 cells were exposed to ELF-MF for 30 min or 48 h.<br>2.Parameters assessed:<br>-changes in $[Ca^{2+}]_i$<br>-ROS and membrane integrity (in cells exposed for 48 h)	•basal or depolarization-evoked $[Ca2+]_i$ - unchanged<br>•ROS level as well as membrane integrity - unchanged

31.	Komaki et al., 2014<br>**Neuroplasticity**	rat<br>(males, adult, n= 10)	50 Hz, 100 μT	1.Rats were exposed 2 h/day for 3 months.<br>2.Parameters assessed (the day after the last exposure)<br>-induction of LTP (excitatory postsynaptic potential, compound action potential)<br>-paired-pulse ratio	•excitatory postsynaptic potential slope ↑<br>•compound action potential ↑<br>•paired-pulse ratio – not changed
32.	Li et al., 2014<br>**Neuroplasticity**<br>**Calcium**<br>**Viability**	dorsal root ganglion neurons	50 Hz, 0.1 mT/1 mT/ 10 mT/100 mT<br>pulsed ELF-MF	1.Cells were exposed to ELF-MF 2 h/day for 1 or 3 days. Cells were treated with calcium channel blockers, calcium stores inhibitors, ERK inhibitor, phospholipase C inhibitors, calcium chelators, calcium store releaser, IP3 blocker, D-AP5 and MK801.<br>2.Parameters assessed:<br>-protein expression of BDNF<br>-gene expression of *Bdnf*<br>-intracellular calcium concentration<br>-cell viability	•gene expression of *Bdnf* ↑ for 1-100 μT after 1 day and for 0,1-100 μT after 3 days<br>•intracellular calcium concentration ↑ for 1 mT after 1 day<br>•cell viability – not changed<br>•gene expression of *Bdnf* and intracellular calcium concentration ↓ after treatment with calcium chelators and calcium channels blocker (1 mT, after 3 days)<br>•gene expression of *Bdnf* ↓ after treatment with ERK inhibitor (1 mT, after 3 days)
33.	Luo et al., 2014<br>**Calcium**	entorhinal cortex neurons	50 Hz, 1 mT/3 mT	1.Cells were exposed to ELF-MF for 24 h (5 min on and 10 min off).<br>2.Parameters assessed:<br>-whole cell currents including high-voltage and low-voltage activated calcium channels<br>-intracellular concentration of calcium	•whole cell currents and high and low-voltage activated calcium channels – not changed<br>•basal levels of the calcium concentration – not changed<br>•calcium concentration ↓ in response to potassium stimulus
34.	Mahdavi et al., 2014<br>**Behaviour**<br>**Stress hormones**	rat<br>(males, n= 8)	1 Hz/ 5 Hz, 0.1 mT	1.Rats were exposed to 1 or 5 Hz 0,1 mT ELF-MF for 1, 3, 7, 14 or 21 days.<br>2.Parameters assessed (after the end of each exposure)<br>-behaviour in open field<br>-plasma levels of glucose, ACTH, CORT and adrenaline	•locomotor activity, rearing and sniffing in open field for 1 Hz – not changed, for 5 Hz ↑<br>•CORT ↓<br>•ACTH ↑<br>•adrenaline ↑ for 1 Hz<br>•glucose ↓ for 1 Hz, and ↑ for 5 Hz
35.	Mattar et al., 2014<br>**Behaviour**	rat<br>(pups aged 2, 4 or 6 weeks, n= 20)	50 Hz, 3.5 mT	1.Pups were exposed to ELF-MF for 6 weeks (1h/day for 6 days).<br>2.Parameters assessed (during and after exposure):<br>-behaviour (activity, inactivity, motion, response to sound, response to light, eating<br>-extended of hair<br>-redness of limbs<br>-protrusion of testis and penis	•during exposure:<br>-activity, motion, response to sound and light ↓<br>-inactivity, redness of limbs, protrusion of testis and penis ↑<br>•after exposure:<br>-activity, motion, response to sound and light, extended of hair (after some time return to normal), redness of limbs (after some time

					return to normal), protrusion of testis and penis (after some time return to normal) ↑<br>-inactivity ↓
36.	Reale et al., 2014<br>**Oxidative stress Viability Immune response**	human SH-SY5Y cells	50 Hz, 1 mT	1.Cell were exposed to ELF-MF for 1, 3, 6 or 24 h. Some cells were co-treated with H_2O_2.<br>2.Parameters assessed:<br>-cell viability,<br>-NOS and CAT activities, $O_2•^-$ production<br>-enzymatic kinetic parameters related to CAT and CYP-450<br>-expression of cyto/chemokines	•cell morphology and viability – not affected<br>•NOS and CAT activities ↑<br>•kinetic parameters characterizing CAT activity- total velocity and rate of decrease in enzyme reaction ↑<br>•CYP-450 activity and $O_2•^-$ production ↑<br>•TGF-β and IL-18BP expression ↑<br>•CAT activity ↓ and ↑ $O_2•^-$ production in cells co-treated with H_2O_2
37.	Ardeshirylajimi and Soleimani, 2015<br>**Calcium Proliferation Genes Viability**	human pluripotent stem cells (iPSCs)	50 Hz, 1.5 mT pulsed ELF-MF	1.Cells were exposed to ELF-MF 8 h/day for 1, 3, 5, 7 or 14 days.<br>2.Parameters assessed:<br>-cell viability, cell division, cell proliferation and mineralization of extracellular matrix<br>-alkaline phosphatase activity intracellular calcium content (after 7 and 14 days of exposure).<br>-Expressions of osteogenesis-related genes: collagen type 1, runt-related transcription factor 2 (Runx2), osteocalcin, osteonectin, alkaline phosphatase, osteoprotegerin, matrix metalloproteinases 1 and 3 (after 7 and 14 days of exposure)	•proliferation ↑<br>•mineralization of the extracellular matrix ↑<br>•calcium content, alkaline phosphatase activity ↑<br>•the gene expression of most osteogenesis-related genes ↑
38.	Chung et al., 2015<br>**Neurotransmitters**	rat (males, n= 10)	60 Hz, 2 mT	1.Rats were exposed to ELF-MF constantly for 2 or 5 days.<br>2.Parameters assessed in cerebellum, cortex, hippocampus, thalamus and striatum:<br>-noradrenaline, vanillylmandelic acid, serotonin, 5-hydroxyindoleacetic acid, dopamine, 3,4-dihydroxyphenylacetic acid, homovanillic acid<br>-aspartic acid, glutamate, glutamine, glycine, taurine, tyrosine, gamma aminobutyric acid<br>-NO	•noradrenaline and vanillylmandelic acid ↑ in all brain structures except of cortex<br>•serotonin ↑ after 5 days in the striatum and thalamus<br>•5-hydroxyindoleacetic acid ↑ in the striatum and hippocampus after 5 days and in thalamus after 2 and 5 days<br>•dopamine ↑ in the thalamus after 5 days<br>•aspartic acid ↓ in the cortex after 5 days<br>•glutamate ↑ in the thalamus after 5 days<br>•glutamine ↓ in the cortex and cerebellum, and ↑ in the striatum and thalamus after 5 days<br>•glycine ↓ in the cortex and hippocampus, and ↑ in the striatum and thalamus after 5 days

					•taurine ↓ in the cortex and hippocampus<br>•tyrosine ↑ in the thalamus after 5 days<br>•gamma aminobutyric acid ↓ in the cortex, cerebellum and hippocampus, and ↑ in the striatum and thalamus<br>•NO ↑ in the stratum after 2 and 5 days, and in the thalamus and hippocampus after 5 days
39.	Duan et al., 2015<br>**DNA damage Viability**	mouse spermatocyte - derived GC-2 cell line	50 Hz, 1 mT/2 mT/ 3 mT	1.Cells were exposed to 50 Hz ELF-MF of magnetic flux densities: 1, 2 and 3 mT for 24 h with intermittency cycles of 5 min field on and 10 min field off.<br>2.Parameters assessed:<br>-cell viability and DNA damages	•cell viability - not affected<br>•DNA strand breaks ↑ (only 3 mT)
40.	Golbach et al., 2015<br>**Calcium**	human neutrophil-like cell lines HL-60 and PLB-985	50 Hz, 5 μT/300 μT/500 μT<br><br>or 50 Hz, 2.5 mT	1.First variant: Cells were exposed to 5, 300 or 500 μT for 30 min (short-term exposure) or subjected to real-time exposure during flow cytometry (50 Hz 2.5 mT).<br>Second variant cells were exposed to 300 μT or 500 μT ELF-MF for 4-5 days.<br>2.Parameters assessed:<br>-intracellular calcium mobilization<br>-genes expression (calcium influx pathway genes).<br>-morphology of HL-60 cells after four-day exposure.	regardless of magnetic flux density value<br>•calcium mobilization – not altered<br>•calcium signaling - unchanged<br>•gene-expression patterns of calcium-signaling related genes- not altered<br>•phenotypic changes – not found
41.	Lewicka et al., 2015<br>**Oxidative stress**	human blood platelets	1 kHz, 0.5 mT<br>50 Hz, 10 mT<br>1 kHz, 220 V/m	1.Human blood platelets were exposed to different sources of electromagnetic radiation: car electronics (1 kHz 0.5 mT), physiotherapy equipment (50 Hz 10 mT) or LCD monitors (1 kHz 220 V/m) for 30 min.<br>2.Parameters assessed (before and after exposure) CAT activity and MDA concentration	•CAT activity and MDA concentration ↑<br>(the most significant changes after exposure to car electronics)
42.	Li et al., 2015<br>**Oxidative stress**	human (males mean age 30.9, females mean age 29.8, n= 310)	Occupational exposure 0.62 – 30.19 μT (500 kV) 0.51 – 60.11 μT (220 kV)	1.Workers occupationally exposed to ELF-MF were included to research.<br>2.Parameters assessed:<br>-plasma levels of TAS and MDA as well as GPx and SOD activities<br>-genotoxicity.	•oxidative stress parameters – not changed<br>•genotoxicity – not induced
43.	Liu et al., 2015<br>**Behaviour**	rat	50 Hz, 400 μT	1.Rats - Alzheimer's Disease Model exposed to ELF-MF 24 h/day for 60 days.	•pathological damages of hippocampus ↓<br>•spatial learning and memory disorder ↓

	**Proteins**	(males, 8 weeks old, n= 16)		1. Parameters assessed:<br>-behaviour in Morris water maze- five consecutive days after exposure<br>-brain damage (15 min/5 days after exposure)<br>-proteomic analysis (15 min after exposure)	•changes in the expression of proteins involved in synaptic transmission (SNCG, SNAP-25b), protein degradation (UCH-L1,UBE2N), oxidative stress (CFL1, PRDX5, PRDX6), energy metabolism (DUSP3, DDT, PDHE1-B, ECH), inflammation (FABP), Tau aggregation (Tpi1, EFHD2), and brain injury (MBP).
44.	Patruno et al., 2015<br>**Oxidative stress**	myelogenous leukemia cell line K562	50 Hz, 1mT	1.Cells were exposed to ELF-MF for 1, 3, 6, 9, 12, 18 or 24 h.<br>2.Parameters assessed:<br>-activities and kinetic parameters of CAT, iNOS and CYP-450	•activities of CAT and CYP-450 ↑<br>•iNOS ↓
45.	Tiwari et al., 2015<br>**Oxidative stress**<br>**Stress hormones**<br>**DNA damage**	human (males, 20-58 years old, n= 142)	occupational exposure to 132 kV high-voltage substations	1.Workers were exposed to ELF-MF for more than 2 years of occupational exposure.<br>2.Parameters assessed:<br>-levels of plasma adrenaline, oxidative stress markers (MDA, NO) and<br>-DNA damages	•adrenaline ↑<br>•DNA damages ↑<br>•MDA and NO ↑
46.	Yang and Ye, 2015<br>**Oxidative stress**<br>**Cell death**<br>**Proliferation**<br>**Viability**	human osteosarcom a MG-63 cells	50 Hz, 1mT	2.Cells were exposed to 1 mT ELF-MF for 1, 2 or 3 h.<br>3.Parameters assessed:<br>-proliferation and apoptosis rate<br>-ROS level<br>-expression of p38MAPK	•cells viability ↓<br>•cell growth ↓<br>•apoptosis ↑<br>•ROS ↑<br>•activation of p38MAPK (kinase implicated in pathological processes)
47.	Zhao et al., 2015<br>**Behaviour**<br>**Neuroplasticity**	ICR mouse (females, 3 to 4 weeks old<br>n = at least 10)	50 Hz, 1 mT	1.Mice were exposed to ELF-MF of 1 mT for 12 h/day for up to 21 days.<br>2.Parameters assessed:<br>-recognition memory ability<br>-locomotor activity<br>-dendritic spine densities of hippocampal CA1 pyramidal cells	•exposure to 1 mT ELF-MF for 7 days:<br>-object recognition ↓<br>-locomotor activity not changed<br>•exposure to 1 mT ELF-MF for 7 or 10 days:<br>-dendritic spine density of neurons in the hippocampus ↓
48.	Falone et al., 2016<br>**Oxidative stress**<br>**Viability**	human neuroblasto ma SH-SY5Y cell line	75 Hz, 2 mT pulsed ELF-MF	1.Cells were exposed to ELF-MF 3 times during 5 days (day 1, 3, 5). 24h after the last exposure cells were treated with H_2O_2 for 10 or 30 min.<br>2.Parameters assessed:<br>-cell viability,	•ELF-MF alone (without H_2O_2) did not affect the oxidative status and viability of the cells<br>•in H2O2 treated cells ELF-MF:<br>-cell viability ↑<br>-ROS level ↓

				-ROS level and MnSOD activity	-MnSOD activity ↑
49.	Ferroni et al., 2016 **Calcium Genes**	human mesenchymal stem cell (MSCs)	100 Hz, less than 40 µT pulsed ELF-MF	1.Cells were cultured in several types of medium: adipogenic, osteogenic, neural or glial differentiative medium and basal medium and then exposed to pulsed ELF-MF 24 min/day for 21 days.<br>2.Parameters assessed:<br>-gene expression of adipogenic, neuronal, glial, and osteogenic markers<br>-expression of angiogenic and mechano-transduction markers (HIF1A, VEGFA, VEGFB, VEGFC, CDC42, RHO).<br>-intracellular lipid content and ALP activity<br>-presence of calcium depots and osteoids	•cells in basal medium:<br>-mRNA level of genes related to angiogenesis ↑<br>-the levels of HIF1A, VEGFA, VEGFB, VEGFC, CDC42, RHO expression ↑<br>-expression of adipogenic, neuronal, glial and osteogenic - unchanged.<br>•cells in adipogenic medium:<br>-intracellular lipid content and adipogenic differentiation markers – not changed<br>•cells in neural/glial differentiative medium<br>-levels of genes involved in neuronal and glial commitment - not changed<br>•cells in osteogenic medium<br>-osteogenic markers ↑<br>-ALP activity ↑<br>-accumulation of calcium depots ↑
50.	de Kleijn et al., 2016<br><br>**Stress hormones**	BalB/c mice (males, 6 weeks old, n= 6-20)	signal contained multiple frequencies 20 Hz-5000 Hz, 10 µT	1.Mice were subjected into short term (1 week) and long term (15 weeks) exposures for 1, 4 or 24 h/day.<br>2.Parameters assessed (after exposure)<br>-leukocyte numbers<br>-hypothalamic CRH, plasma ACTH, pituitary POMC, expression of CYP11A1 in adrenal glands (short term exposure)	•effect only short-term exposure (1 week):<br>-number of leukocytes ↑ (24h/day)<br>-CRH – not affected<br>-ACTH ↓ (4 h/day)<br>-POMC ↓ (24 h/day)<br>-CYP11A1 expression – not altered
51.	Lai et al., 2016 **Behaviour**	rat (males, adult, n= 10)	50 Hz, 100 µT	1.Rats were exposed to ELF-MF 20 h/day for 24 weeks.<br>2. Parameters assessed: after exposure<br>-behaviour in open field, Morris water maze, tail suspension test, elevated plus maze, forced swim test, fear conditioning test<br>- brain morphology and histology.	•behaviour - not affected<br>•brain morphology and histology - not affected
52.	Luo et al., 2016 **Oxidative stress**	ICR mouse (males, 3 weeks old, n= 12)	50 Hz, 8 mT	1.Mice were exposed to ELF-MF 4 h/day for 28 days.<br>2.Parameters assessed (after the last exposure)<br>-SOD, CAT, GPx, GR and GST activities and MDA level in blood and cerebral cortex	•antioxidant enzymes ↓<br>•MDA level ↑
53.	Nakayama et al., 2016	macrophage RAW264 cells	50 Hz, 0.5 mT	1.Cells were exposed to ELF-MF for 24 h.<br>2.Parameters assessed:<br>-DNA damages	•cell viability - not affected<br>•NO production – not affected<br>•DNA damage – not found

	**Oxidative stress**<br>**DNA damage**<br>**Viability**			-cell viability<br>-NO level	
54.	Sun et al., 2016<br>**Calcium**<br>**Neuroplasticity**	C57 mouse (males, females, pups,<br>n= 4-30)	50 Hz, 1 mT	1.Young mice were exposed to ELF-MF from birth to 8-10 postnatal days.<br>2.Parameters assessed (after exposure)<br>-protein and gene expression of calcium ion channels (total, P/Q, N and R subtypes)<br>-exocytosis<br>-frequency and amplitude of excitatory postsynaptic currents<br>-size of the readily releasable pool<br>-slow and rapid endocytosis of synaptic vesicles<br>-endocytosis overshoot<br>-bulk endocytosis<br>-post-tetanic potentiation	•calcium influx ↑<br>•endocytosis ↑<br>•no effect on the size of the readily releasable pool or exocytosis<br>•post-tetanic potentiation ↑<br>•protein and gene expression of P/Q and N subtypes of calcium channels ↑
55.	Wei et al., 2016<br>**Cell death**<br>**Calcium**<br>**Proteins**	cardiomyocytes isolated from neonatal rats	15 Hz, 2 mT	1.Cells were exposed to ELF-MF for 30 min and incubated in hypoxic conditions for 12 h (ELF-MF+hypoxia), part of cells was pre-treated with Hsp70 inhibitor and exposed to the same conditions.<br>2.Parameters assessed:<br>-Morphology<br>-protein content<br>-cytotoxicity<br>-concentration of free intracellular calcium<br>-expression of *Hsp70*<br>-expression of pro-apoptotic proteins (Bax, capsase-3)<br>-expression of anti-apoptotic protein Bcl-2	•effects of ELF-MF on cells under hypoxic conditions:<br>-cytotoxicity ↓<br>-protein content ↓<br>-expression of Bax and caspase-3 ↓<br>-expression of Bcl-2 ↑<br>-hypertrophy ↓<br>-$[Ca^{2+}]_i$ oscillation baseline ↓<br>-amplitude of $[Ca^{2+}]_i$ oscillation ↑<br>-expression of *Hsp70* ↑<br>•Hsp70 inhibitor suppresses ELF-MF induced-cardioprotection
56.	Wyszkowska et al., 2016<br>**Behaviour**<br>**Proteins**	*Schistocerca gregaria* desert locust (males/females, adult 4-9 days post-moult, n= 36)	50 Hz, 1 mT/4 mT/7 mT	1.Insects were exposed to ELF-MF for 24 h.<br>2. Parameters assessed:<br>-walking behaviour and muscle force dynamics<br>-action potential and excitatory postsynaptic potential (EPSP) properties of the fast extensor tibiae motor neuron (FETi)<br>-Hsp70 level	•motor activity ↓ (4 and 7 mT)<br>•muscle force ↓<br>•latency and duration of the FETi action potential ↑ (7 mT)<br>•Hsp70 ↑ (7 mT)

57.	Yin et al., 2016 **Oxidative stress Cell death DNA damage Viability Calcium**	rat hippocampal neurons	50 Hz, 8 mT	1.Cells were exposed to ELF-MF for 90 min.<br>2.Parameters assessed:<br>-cell viability and morphology<br>-MDA level and SOD activity<br>-intracellular Ca^{2+} level<br>-mitochondrial membrane potential<br>-intracellular ROS level<br>-DNA damages<br>-cell cycle and apoptosis	•cell viability ↓<br>•morphologically - changed,<br>•SOD activity ↓<br>•MDA and ROS levels ↑<br>•Ca^{2+} level ↑<br>•depolarization of mitochondrial membrane potential ↑<br>•apoptosis and DNA damages ↑<br>•number of cells in G0/G1 phase ↓
58.	Zhang et al., 2016 A **Oxidative stress DNA damage**	human (n= 190)	ELF-MF of 110-420 kV	1.Workers exposed to high-voltage power lines.<br>2.Parameters assessed:<br>oxidative stress (8-isoprostane)<br>oxidative damage to DNA (8-hydroxy-2-deoxy-guanosine, 8-OHdG)<br>in urine	•8-isoprostane and 8-OhdG levels ↑
59.	Zhu et al., 2016 **DNA damage**	human lens epithelial cells	50 Hz, 0.4 mT	1.Cells were exposed to ELF-MF for 2, 6, 12, 24 or 48 h.<br>2.Parameters assessed:<br>-DNA damages	•DNA fragmentation – not found
60.	Calcabrini et al., 2017 **Oxidative stress**	human keratinocyte cell line NCTC 2544	50 Hz, 25 µT- 200 µT	1.Cells were exposed to ELF-MF for 1, 2 or 4 h.<br>2.Parameters assessed:<br>-ROS production<br>-GSH content, lipid peroxidation and antioxidant defense activity (after 1, 2 and 4 h of exposure to 50 and 100 µT)	•ROS production (1 h, 50 and 100 µT) ↑<br>•GSH content ↓ (1 and 2 h of) ↑ (4 h)<br>•antioxidant enzymes activities (SOD, GPx, GR) ↓<br>•TBARS level ↑
61.	Cichoń et al., 2017 **Oxidative stress Behaviour**	post stroke patients (mean age 68 years old, n= 23	40 Hz, 7 mT various pulse shape quantities	1.Post stroke patients subjected to 15 min therapy 5 days/week for 4 weeks.<br>2.Parameters assessed:<br>-CAT, SOD activities<br>-plasma TAS<br>-functional and mental status	•CAT, SOD activities ↑<br>•TAS level - not changed<br>•functional and mental status ↑
62.	Djordjevic et al., 2017 **Oxidative stress Behaviour**	rat (males, 3 months old, n= 5)	50 Hz, 10 mT	1.Rats were exposed to ELF-MF 24 h/day for 7 days.<br>2. Parameters assessed (24 h after the last exposure)<br>-behaviour in open field and elevated plus maze<br>-concentrations of $O_2{\bullet}^-$, NO_2^- and $ONOO^-$ in hypothalamic tissue	•activity in elevated plus maze and open field ↓<br>•$O_2{\bullet}^-$ and NO_2^- ↑<br>•$ONOO^-$ - not changed

63.	Ehnert et al., 2017 **Oxidative stress**	human osteoblasts	16 Hz, 6-282 μT pulsed ELF-MF	1. Osteoblasts were exposed to pulsed ELF-MF for 7 min (single or repetitive exposure 7 min/day for 5 days (>3).<br>2. Parameters assessed:<br>-formation of $O_2{\bullet}^-$, H_2O_2, HO•, $ONOO^-$, GSH content<br>-expression of *SOD1*, *SOD2*, *CAT*, *GPX1, GPX3, GPX4* and *GSR*<br>-SOD2, CAT, GPXs and GSR proteins	•single exposure:<br>-$O_2{\bullet}^-$ and H_2O_2 ↑,<br>-HO•, $ONOO^-$ and GSH content – not affected<br>•repetitive exposure to ELF-MF:<br>-ROS production ↓<br>-*SOD2*, *CAT*, *GPX3,* and *GSR* ↑
64.	Haghighat et al., 2017 **Proliferation Proteins Genes**	rat bone marrow mesenchymal stem cells (BMSC)	50 Hz, 20 mT	1.Cells were exposed to ELF-MF for 1 week.<br>2.Parameters assessed:<br>-expression of genes responsible for pluripotency and neuronal differentiation as well as their proteins level<br>-cell morphology	•cell proliferation ↓<br>•cell length and multi-polarization of cells ↑ (neurons' morphology)<br>•expression of genes responsible for pluripotency and neuronal differentiation ↓
65.	Kuzay et al., 2017 **Oxidative stress**	rat (males, adult, n= 6)	50 Hz, 8.2 mT	1.Rats – diabetic model were exposed 20 min/day for 1 month for 5 days a week.<br>2.Parameters assessed (after the last exposure)<br>-testicular tissue levels of MDA, total NO and GSH	•MDA and NO level ↑<br>•GSH level ↓
66.	Sakhaie et al., 2017 **Neuroplasticity Behaviour**	mouse (males, 6-7 weeks old, n=14)	50 Hz, 1 mT	1.Neurotoxin-injected mice were exposed to ELF-MF 6h/day for 6 days.<br>2.Parameters assessed:<br>-neuronal maturation (24 h after the last exposure)<br>-neurogenesis (24 h after the last exposure)<br>-memory (Morris water maze test) (32 days after exposure)	ELF-MF reversed:<br>•learning and memory abilities impairment<br>•neuronal maturation impairment<br>•neurogenesis decrease
67.	Urnukhsaikhan et al., 2017 **Neuroplasticity Cell death Immune response Behaviour**	C57B6 mice (males, 8 weeks old, n= 18)	60 Hz, 10 mT pulsed ELF-MF	1.Ischemic mice were exposed to ELF-MF 6 h/day for 14 days.<br>2.Parameters assessed:<br>-motor abilities (rotarod tests, 3, 7, 11, and 14 days after surgery) After 1, 3 or 14 days<br>-expression of anti-apoptotic *Bcl-xL* and pro-apoptotic *Bax* and *Bad*<br>-expression of Bad, phosphorylated Bad, Bax, caspase 3, Bcl-xL,<br>-BDNF, TrkB, PKB, phosphorylated PKB<br>-inflammation related MMP9, IL-1β and IL-6β infarct volume	•motor abilities ↑<br>•expression of BDNF, TrkB and phosphorylated PKB ↑<br>•expression of pro-apoptotic Bad, Bax and caspase 3 ↓<br>•expression of anti-apoptotic Bcl-xL ↑<br>•inflammatory mediators MMP9 and IL-1β ↓
68.	Budziosz et al., 2018	rat	50 Hz, 10 kV/m, 4.3 pT	1.Rats were exposed to ELF-MF 22 h/day for 28 days	•MDA level and TOS in central nervous system - not changed

	**Oxidative stress**	(males, 10 weeks old, n= 10)		2.Parameters assessed (24 h after the last exposure)<br>-TOS, MDA, SOD and its isoenzymes (CuZnSOD, MnSOD), CAT, GPx, GR, GST and TAC	•activities of antioxidant enzymes ↓(except for frontal cortex CAT, GPx and hippocampal GR)<br>•non-enzymatic antioxidants – not affected (except the frontal cortex).
69.	Burman et al, 2018 **Behaviour**	BALB/cAnNCrl mouse C57BL/6NCrl mouse (females, juvenile 6-8 weeks old, n= 5)	5-100 Hz	1.Mice were exposed to ELF-MF in 6 week period.<br>2.Parameters assessed:<br>-food and water uptake (every week)<br>-indirect behavioural and physical measures: injury/wound scores [present/absent]; barbering score [present/absent]; whisker trimming score [present/absent], body weight (g) (every week)<br>-position of mice within the cage (every week)<br>-behaviour in open field test and novel-object recognition test (after the end of the exposure)	•no effect found
70.	Cichoń et al., 2018 A **Oxidative stress**	human (males, females, mean age 68 years old, n= 23)	40 Hz, 5 mT various pulse shape quantities	1.Post-stroke patients were subjected to magnetotherapy (15 min x 20 treatments).<br>Parameters assessed:<br>-protein carbonyl groups, thiol groups, MDA level	•carbonyl groups and MDA levels ↓<br>•thiol groups level ↑
71.	Cichoń et al., 2018 B **Neuroplasticity Immune response Behaviour**	post stroke patients (mean age 48 years old, n= 25)	40 Hz, 5 mT various pulse shape quantities	1.Post stroke patients subjected to 15 min therapy, 10 sessions with an interval of 14 days.<br>2.Parameters assessed in plasma:<br>-BDNF, Expression of *BDNF,* VEGF level<br>-cytokines: HGF, SCF, SDF-1α, β-NGF and LIF<br>-neurologic deficits<br>-functional and cognitive status<br>-level of depression	•level of BDNF ↑<br>•expression of *BDNF* ↑<br>•level of VEGF ↑<br>•HGF and SCF levels ↑<br>•SDF-1α level – not changed<br>•neurologic deficits ↓<br>•functional and cognitive status ↑<br>•depressive syndrome ↓
72.	Fathi and Farahzadi, 2018 **Proliferation**	rat adipose tissue-derived mesenchymal stem cells (rADSCs)	50 Hz, 20 mT	1.rADSCs were first cultured in adipogenic, osteogenic, chondrogenic and neurogenic mediums and exposed to ELF-MF 30 min/day for 21 days.<br>2.Parameters assessed:<br>-cell proliferation<br>-population double time	•cell proliferation ↓<br>•population double time - prolonged
73.	Laszlo et al., 2018	turkey (females, adult, n= 40)	50 Hz, 10 µT pulsed ELF-MF	1.Turkeys were exposed 20 min every 8 h for 3 weeks.<br>2.Parameters assessed:	•activity ↓<br>•hemoglobin, SGOT, SGPT, AP, γGT and LDH – not changed

	**Behaviour**<br>**Stress hormones**			-the behaviour (relaxation, play, competition, aggression).<br>-hemoglobin, SGOT, SGPT, AP, γGT, LDH (blood , every week)<br>-level of cAMP to detect noradrenaline-activated β-adrenoreceptor function (blood , every week)	•noradrenaline-activated β-adrenoceptor function ↓
74.	Martínez-Sámano et al., 2018<br>**Oxidative stress**<br>**Stress hormones**<br>**Lipids**	rat (males, 8 weeks old, n= 6)	60 Hz, 2.4 mT	1.Rats (restrained (ELF-MF+RS) and unrestrained (ELF-MF)) were exposed to ELF-MF for 2 h/day for 21 days.<br>2.Parameters assessed (after the last exposure)<br>-total cholesterol, and triacylglycerol levels, TBARS<br>-plasma CORT concentrations,<br>-total free fatty acids and fatty acid methyl esters (FAMEs)	•ELF-MF group:<br>-total cholesterol and triacylglycerol levels not affected<br>-CORT level ↑<br>-total lipids in cerebellum and total cholesterol in cortex ↑<br>-polar lipids in cortex ↓<br>-polyunsaturated fatty acids in cerebellum ↓ and ↑ in subcortical structures.<br>•TBARS in total lipids ↑ in both groups<br>•concentrations of non-esterified fatty acids in subcortical structures (RS+ELF-MF) ↑
75.	Rezaie-Tavirani et al, 2018<br>**Proteins**	rat (males, adult, n= not provided)	50 Hz, 0.5 mT/ 1 mT	1.Rats were exposed to 0.5 mT or 1 mT 3 h/day for 2 or 4 weeks.<br>2.Parameters assessed:<br>-proteome profile<br>-protein-protein interaction<br>-molecular function	•0.5 mT:<br>-64 spots up-regulated<br>-40 spots down-regulated<br>•1 mT:<br>-86 spots up-regulated<br>-65 spots down-regulated<br>•expression of Sptan1 and Dpysl2 ↓ by 0.5 mT<br>•expression of Dpysl2 ↑ by 1 mT after 2 weeks<br>•expression of Tpi1 and Lap3 ↓ through time<br>•expression Tppp ↓ by 1 mT through time<br>•general protein expression ↓ with increasing ELF-MF intensity and time<br>•identified proteins are related to apoptosis, stress response and number of metabolic processes.
76.	Song et al., 2018<br>**Oxidative stress**<br>**Proliferation**	HeLa cells<br>IMR-90 fibroblasts	60 Hz, 3 mT/ 6 mT	Variants of experiments:<br>1.Cells were exposed to 6 mT ELF-MF for 30 or 60 min.	•single exposure to 6 mT and repetitive exposure to 3 or 6 mT:<br>-DNA damages and change of cell viability - not found.<br>•exposure to 6 mT for total 168 h:

	**DNA damage**<br>**Viability**			2.Cells were exposed to 3 and 6 mT ELF-MF 30 min every 24 h for 72 h or 30 min 8 times a day for 3 days.<br>3.Cells exposed to 6 mT for 72 h were additionally exposed for subsequent 96 h.<br>Parameters assessed:<br>DNA damage, cell viability<br>cell cycle and ROS level (during continuous exposure)<br>4.Cells were co-incubated with GOx, (to induce H_2O_2 and reduce proliferation) and exposed to ELF-MF for 1, 6, 12 and 24 h<br>Parameters assessed:<br>phosphorylation of PKB and Erk1/2	-cells viability and cell cycle progression ↑<br>-ROS level ↓<br>•in GOx – treated cells, ELF-MF:<br>-mitigated anti-proliferative effect<br>-phosphorylation of PKB and ERK1/2 ↑
77.	Sun et al., 2018<br>**Oxidative stress**	*Caenorhabdit is elegans*<br>(n= 90300)	50 Hz, 3 mT	1.Worms were exposed to ELF-MF from egg stage reaching the fourth larva (L4) stage (about 48 h).<br>2.Parameters assessed:<br>-expression of the genes involved in TCA cycle (associated with tumor growth). PGE_2, ROS, TAC level<br>-SOD and CAT activities	•TCA cycle enzyme ↓<br>•arachidonic acid and PGE_2 ↑<br>•expression of PGE_2 synthase ↑<br>•ROS level ↑<br>•TAC level ↓<br>•SOD and CAT activities – not changed
78.	Wyszkowska et al., 2018<br>**Immune response**	rat<br>(males, adults, n= 6)	50 Hz, 7 mT	1.Rats were subjected to acute (24 h) or repetitive exposure (1 h/day for 7 days).<br>2.Parameters assessed (plasma):<br>-cytokines: IL-1β, IL-2, IL-6, IL-10<br>-number of: total white blood cells, lymphocytes, monocytes, granulocytes red blood cells, platelets<br>-hemoglobin<br>-Hematocrit	•24 h exposure:<br>-IL-1β, IL-2, IL-6 ↑<br>-white blood cells, lymphocytes, red blood cells, hemoglobin and hematocrit ↑<br>•repetitive exposure 1 h/day for 7 days – no effect
79.	Cichoń et al., 2019<br>**Immune response**	post stroke patients<br>(mean age 44,8 years old,<br>n= 25)	40 Hz, 5 mT pulsed ELF-MF	1.Post stroke patients subjected to 15 min therapy, 10 sessions with an interval of 14 days.<br>2.Parameters assessed:<br>-Plasma levels of cytokines: IL-1β, IL-2, IFN-γ, TGF-β<br>-Expression of *IL-1β*	•level of IL-1β ↑<br>•expression level of *IL-1β* ↑<br>•level of IL-2 ↑<br>•IFN-γ and TGF-β levels – not changed
80.	Hosseinabadi and Khanjani, 2019	human<br>(20-50 years old, n= 152)	Mean:<br>4,09 V/m,<br>16.27 μT	1.Power plant workers (occupational exposure)<br>2.Parameters assessed:<br>-serum TAC, MDA, SOD, CAT and GPx	•oxidative stress ↑

	**Oxidative stress**				
81.	Hosseinabadi et al., 2019 **Behaviour**	human (30-40 years old, n= 132)	occupational exposure	1.Participants were exposed to ELF-MF (the 8-h time-weighted average).<br>2.Parameters assessed:<br>-sleep quality<br>-depression, anxiety and stress levels	•sleep quality ↓<br>•stress, depression and anxiety ↑ (linear relation (trend) with increased exposure)
82.	Karimi et al., 2019 **Oxidative stress Behaviour**	rat (males, adult, n= 12)	50 Hz, 1 μT/100 μT/500 μT/ 2000 μT	1.Rats were exposed to ELF-MF 2 h/day for 60 days.<br>2.Parameters assessed:<br>-elevated plus maze (the day after the last exposure) and Morris water maze test (the day after elevated plus maze)<br>-the passive avoidance test (one week after Morris water maze)<br>-MDA, TAC, TOS and total thiol molecules (after behavioural tests)	•lipid peroxidation ↑ (100 μT and 500 μT)<br>•TAC ↑ (1 μT and 500 μT)<br>•total thiol molecules ↑ (all induction values)<br>•TOS ↑ (500 μT).<br>•memory retention ↑ (100 μT and 2000 μT, water maze)<br>•memory retention ↑ (100 μT, 500 μT and 2000 μT, passive avoidance test)<br>•anxiety ↑ (all induction values)
83.	Merla et al., 2019 **Oxidative stress**	SH-SY5Y neuroblastoma cells	50 Hz, 1 mT	1.Cells were treated with DPI an inhibitor of the plasma membrane enzyme NADPH oxidase (Nox) and then exposed for ELF-MF for 24 h.<br>1.Parameters assessed:<br>-source of ROS production	•ROS generation ↑<br>•plasma membrane Nox is involved in redox imbalance elicited by ELF-MF
84.	Sun et al., 2019 **Proteins Genes Lipids**	*Caenorhabditis elegans* (n= 40000)	50 Hz, 3 mT	1.Worms were placed under 3 mT ELF-MF till reaching the L4 stage (48 h).<br>2.Parameters assessed:<br>-total triacylglycerols (TGs) level (proteomic and transcriptomic profiling)	•in glycerolipids (GLs) category:<br>-total triacylglycerols (TGs) ↑<br>-diacylglycerols (DGs) ↓<br>•other identified lipid categories showed no regular pattern of changes<br>-stress response related genes expressions ↑<br>-the most enriched protein functions were: defense response, reproduction and lipid transport
85.	Mahaki et al., 2020 **Immune response**	rat (males, adults, n= 16)	50 Hz, 1 μT/100 μT/500 μT/2000 μT	1.Rats were exposed to ELF-MF 2h/day for 60 days at two phases: at pre- and post-stimulation of the immune system.<br>2.Parameters assessed:<br>-serum levels of cytokines: IL-9, IL-10, TNF-α	•before immunization:<br>-IL-9 ↓ for 100 μT<br>-TNF-α ↓ for 100 μT-2000 μT<br>-IL-10 ↑ for 1 μT and 100 μT<br>•after immunization:<br>-IL-9, TNF-α ↓ for 1 μT and 100 μT<br>-no significant differences in IL-10 level

					•post-immunization levels compared to pre-immunization:<br>-IL-9, TNF-α ↑ for 100 μT-2000 μT<br>-IL-10 ↑ for 2000 μT
86.	Touitou et al., 2020<br>**Proteins**	human (males, mean 38 years old, n= 15)	50 Hz, mean 0.9 μT	1.Men chronically exposed to ELF-MF (1-20 years).<br>2.Parameters assessed:<br>-serum level of CgA	•CgA levels ↓ in participants exposed to highest level of exposure.

Abbreviations: 8-OHdG- 8-hydroxy-2'-deoxyguanosine; **$[Ca]^{2+}_i$** – intracellular ionized calcium; **ACTH** - adrenocorticotropic hormone; **ALP**- alkaline phosphatase; **ALT**- alanine transaminase; **AP**- alkaline phosphatase; **AST**- aspartate transaminase; **Bad**- Bcl-2-associated death promoter; **Bax**- Bcl-2-associated X protein; **Bcl-2**- B cell leukemia/lymphoma-2; **Bcl-xL**- B-cell lymphoma-extra-large; **BDNF**- brain derived neurotrophic factor; **cAMP**- 3′5′-cyclic-adenosine-monophosphate; **CAT**- catalase; **CDC42**- cell division control protein 42 homolog; **CFL1**- cofilin 1; **CgA**- chromogranin A; ***CHEK1***- checkpoint kinase 1 encoding gene; **CORT**- corticosterone/cortisol; **CRH**- corticotropin-releasing hormone; **CuZnSOD (SOD3)**- copper-zinc-superoxide dismutase; **CYP11A1**- cytochrome P450 11A1; **CYP17A1**- cytochrome P450 17A1/steroid 17α-monooxygenase; **CYP-450**- cytochromes P450; **D-AP5**- 5-phosphono-D-norvaline; **DDT**- D-dopachrome decarboxylase; ***DKK1***- dickkopf-related protein 1 encoding gene; **DPI**- diphenyleneiodonium; **Dpysl2**- dihydropyrimidinase-related protein 2; **DUSP3**- dual specificity phosphatase 3; **ECH**- 2-Enoyl-Coa Hydratase; **EFHD2**- EF-hand domain-containing protein D2; **ERK**- extracellular signaling-regulated kinase; **FABP**- fatty acid-binding protein; **free-T3**- free triiodothyronine; **free-T4**- free thyroxine; **GDNF**- glial cell-derived neurotrophic factor; **GOx**- glucose oxidase; **GPx**- glutathione peroxidase; **GR**- glutathione reductase; **GSH**- glutathione; **GSR**- glutathione-disulfide reductase; **GST**- glutathione S-transferase; **HGF**- hepatocyte growth factor; **HIF-1**- hypoxia-inducible factor 1; **HIF-1A**- hypoxia-inducible factor 1-alpha; **HO•** - hydroxyl radicals; **Hsp70**- heat shock protein 70; **IFN-γ**- interferon-γ; **IGF-1**- insulin-like growth factor 1; **IL-10**- interleukin 10; **IL-18BP**- interleukin-18-binding protein; **IL-1β**- interleukin 1β; **IL-2**- interleukin 2; **IL-6**- interleukin 6; **IL-6β**- interleukin 6β; **iNOS**- inducible nitric oxide synthase; **IP3**- inositol trisphosphate; **Lap3**- cytosol aminopeptidase; **LDH**- lactate dehydrogenase; **LIF**- leukemia inhibitory factor; **LTP**- long-term potentiation; **MBP**- myelin basic protein; **MDA**- malondialdehyde; **MK801**- dizocilpine; **MMP9**- matrix metallopeptidase 9; **MnSOD (SOD2)**- manganese-dependent superoxide dismutase; **MPO**- myeloperoxidase; ***NDRG4***- N-myc downregulated gene 4; **NO**- nitric oxide; **NO_2^-** - nitrite; **NOS**- nitric oxide synthase; **$O_2•^-$** -superoxide anion; **$ONOO^-$** - peroxynitrite; **OSI**- oxidative stress index; **p38MAPK**- p38 mitogen-activated protein kinase; **PDHE1-B**- pyruvate dehydrogenase E1; **PGE_2** – prostaglandin E_2; **PI3K**- phosphoinositide 3-kinase; **PKB**- protein kinase B; **POMC**- pro-opiomelanocortin; **PP2A**- protein phosphatase 2; **PRDX5**- peroxiredoxin-5; **PRDX6**- peroxiredoxin-6; **RHO**- ras homolog family; **ROS**- reactive oxygen species; **rTMS**- repetitive transcranial magnetic stimulation; **S100-A10**- S100 calcium-binding protein A10; **S100-A11**- S100 calcium-binding protein A11; **SCF**- stem cell factor; **SDF-1α**- stromal derived factor-1α; **SGOT**- serum glutamic-oxaloacetic transaminase; **SGPT**- serum glutamic-pyruvic transaminase; **SNAP-25b**- synaptosomal-associated protein 25b; **SNCG**- synuclein gamma; **SOD**- superoxide dismutase; ***SPRR3***- small proline-rich protein 3 encoding gene; **Sptan1**- spectrin alpha chain; **TAC**- total antioxidant capacity; **TAS**- total antioxidant status; **TBARS**- thiobarbituric acid reactive substances; **TCA cycle**- tricarboxylic acid cycle/ the Krebs cycle; **TGF-β**- transforming growth factor β; **TOS**- total oxidant status; **Tpi1**- triose phosphate isomerase; **Tppp**- tubulin polymerization-promoting protein; **TrkB**- tyrosine receptor kinase B; **UBE2N**- ubiquitin-conjugating enzyme E2 N; **UCH-L1**- ubiquitin carboxy-terminal hydrolase L1; **VEGF**- vascular endothelial growth factor; **VEP**- visual evoked potential; **β-NGF**- β nerve growth factor; **γGT**- gamma-glutamyl transpeptidase

References

1. Akdag, M.Z.; Dasdag, S.; Ulukaya, E.; Uzunlar, A.K.; Kurt, M.A.; Taşkin, A. Effects of extremely low-frequency magnetic field on caspase activities and oxidative stress values in rat brain. Biol Trace Elem Res 2010, 138(1-3), 238-249; doi: 10.1007/s12011-010-8615-3
2. Cuccurazzu, B.; Leone, L.; Podda, M.V.; Piacentini, R.; Riccardi, E.; Ripoli, C.; Azzena, G.B.; Grassi, C. Exposure to extremely low-frequency (50 Hz) electromagnetic fields enhances adult hippocampal neurogenesis in C57BL/6 mice. Exp Neurol 2010, 226(1), 173-82; doi: 10.1016/j.expneurol.2010.08.022
3. El-Helaly, M.; Abu-Hashem, E. Oxidative stress, melatonin level, and sleep insufficiency among electronic equipment repairers. Indian J Occup Environ Med 2010, 14(3), 66-70; doi:10.4103/0019-5278.75692
4. Frahm, J.; Mattsson, M.O.; Simkó, M. Exposure to ELF magnetic fields modulate redox related protein expression in mouse macrophages. Toxicol Lett 2010, 192(3), 330-336; doi: 10.1016/j.toxlet.2009.11.010
5. Garip, A.I.; Akan, Z. Effect of ELF-EMF on number of apoptotic cells; correlation with reactive oxygen species and HSP. Acta Biol Hung 2010, 61(2), 158-167; doi: 10.1556/ABiol.61.2010.2.4
6. George, M.S.; Lisanby, S.H.; Avery, D.; McDonald, W.M.; Durkalski, V.; Pavlicova, M.; Anderson, B.; Nahas, Z.; Bulow, P.; Zarkowski, P.; Holtzheimer, P.E. 3rd; Schwartz, T.; Sackeim, H.A. Daily left prefrontal transcranial magnetic stimulation therapy for major depressive disorder: a sham-controlled randomized trial. Arch Gen Psychiatry 2010, 67(5), 507-516; doi: 10.1001/archgenpsychiatry.2010.46
7. Goraca, A.; Ciejka, E.; Piechota, A. Effects of extremely low frequency magnetic field on the parameters of oxidative stress in heart. J Physiol Pharmacol 2010, 61(3), 333-338
8. Mannerling, A.C.; Simkó, M.; Mild, K.H.; Mattsson, M.O. Effects of 50-Hz magnetic field exposure on superoxide radical anion formation and HSP70 induction in human K562 cells. Radiat Environ Biophys 2010, 49(4), 731-741; doi: 10.1007/s00411-010-0306-0
9. Martínez-Sámano, J.; Torres-Durán, P.V.; Juárez-Oropeza, M.A.; Elías-Viñas, D.; Verdugo-Díaz, L. Effects of acute electromagnetic field exposure and movement restraint on antioxidant system in liver, heart, kidney and plasma of Wistar rats: a preliminary report. Int J Radiat Biol 2010, 86(12), 1088-1094; doi: 10.3109/09553002.2010.501841
10. Morabito, C.; Rovetta, F.; Bizzarri, M.; Mazzoleni, G.; Fanò, G.; Mariggiò, M.A. Modulation of redox status and calcium handling by extremely low frequency electromagnetic fields in C2C12 muscle cells: A real-time, single-cell approach. Free Radic Biol Med 2010, 48(4), 579-589; doi: 10.1016/j.freeradbiomed.2009.12.005
11. Szemerszky, R.; Zelena, D.; Barna, I.; Bárdos, G. Stress-related endocrinological and psychopathological effects of short- and long-term 50Hz electromagnetic field exposure in rats. Brain Res Bull 2010, 81(1), 92-99; doi: 10.1016/j.brainresbull.2009.10.015
12. Emre, M., Cetiner, S.; Zencir, S.; Unlukurt, I.; Kahraman, I.; Topcu, Z. Oxidative stress and apoptosis in relation to exposure to magnetic field. Cell Biochem Biophys 2011, 59(2), 71-77; doi: 10.1007/s12013-010-9113-0
13. He, L.H.; Shi, H.M.; Liu, T.T.; Xu, Y.C.; Ye, K.P.; Wang, S. Effects of extremely low frequency magnetic field on anxiety level and spatial memory of adult rats. Chin Med J (Engl) 2011, 124(20), 3362-3366
14. Hosseini, E.; Nafisi, S.; Zare, S. The effects of electromagnetic fields on plasma levels of corticosterone, free-T3, free-T4 malonyldialdehyde in white male rabbit with normal diet and hyperchlostrol diet, Veterinary Research Forum 2011, 2(4), 222- 225
15. Juszczak, K.; Kaszuba-Zwoinska, J.; Thor, P.J. Pulsating electromagnetic field stimulation of urothelial cells induces apoptosis and diminishes necrosis: new insight to magnetic therapy in urology. J Physiol Pharmacol 2012, 63(4), 397-401

16. Kirschenlohr, H.; Ellis, P.; Hesketh, R.; Metcalfe, J. Gene expression profiles in white blood cells of volunteers exposed to a 50 Hz electromagnetic field. Radiat Res 2012, 178(3), 138-149; doi: 10.1667/rr2859.1
17. Kitaoka, K.; Kitamura, M.; Aoi, S.; Shimizu, N.; Yoshizaki, K. Chronic exposure to an extremely low-frequency magnetic field induces depression-like behavior and corticosterone secretion without enhancement of the hypothalamic-pituitary-adrenal axis in mice. Bioelectromagnetics 2013, 34(1), 43-51; doi: 10.1002/bem.21743
18. Korpinar, M.A.; Kalkan, M.T.; Tuncel, H. The 50 Hz (10 mT) sinusoidal magnetic field: effects on stress-related behavior of rats. Bratisl Lek Listy 2012, 113(9), 521-524; doi: 10.4149/bll_2012_117
19. Martínez-Sámano, J.; Torres-Durán, P.V.; Juárez-Oropeza, M.A.; Verdugo-Díaz, L. Effect of acute extremely low frequency electromagnetic field exposure on the antioxidant status and lipid levels in rat brain. Arch Med Res 2012 , 43(3), 183-9; doi: 10.1016/j.arcmed.2012.04.003
20. Tasset, I.; Medina, F.J.; Jimena, I.; Agüera, E.; Gascón, F.; Feijóo, M.; Sánchez-López, F.; Luque, E.; Peña, J.; Drucker-Colín, R.; Túnez ,I. Neuroprotective effects of extremely low-frequency electromagnetic fields on a Huntington's disease rat model: effects on neurotrophic factors and neuronal density. Neuroscience 2012, 209, 54-63; doi: 10.1016/j.neuroscience.2012.02.034
21. Vannoni, D.; Albanese, A.; Battisti, E.; Aceto, E.; Giglioni, S.; Corallo, C.; Carta, S.; Ferrata, P.; Fioravanti, A.; Giordano, N. In vitro exposure of human osteoarthritic chondrocytes to ELF fields and new therapeutic application of musically modulated electromagnetic fields: biological evidence. J Biol Regul Homeost Agents 2012, 26(1), 39-49
22. Yang, Y.; Li, L.; Wang, Y.G.; Fei, Z.; Zhong, J.; Wei, L.Z.; Long, Q.F.; Liu, W.P. Acute neuroprotective effects of extremely low-frequency electromagnetic fields after traumatic brain injury in rats. Neurosci Lett 2012, 516(1), 15-20; doi: 10.1016/j.neulet.2012.03.022
23. Amaroli, A.; Chessa, M.G.; Bavestrello, G.; Bianco, B. Effects of an extremely low-frequency electromagnetic field on stress factors: a study in Dictyostelium discoideum cells. Eur J Protistol 2013, 49(3), 400-405; doi:10.1016/j.ejop.2012.12.002
24. Collard, J.F.; Lazar, C.; Nowé, A.; Hinsenkamp, M. Statistical validation of the acceleration of the differentiation at the expense of the proliferation in human epidermal cells exposed to extremely low frequency electric fields. Prog Biophys Mol Biol 2013, 111(1), 37-45; doi: 10.1016/j.pbiomolbio.2012.12.004
25. Corallo, C.; Battisti, E.; Albanese, A.; Vannoni, D.; Leoncini, R.; Landi, G.; Gagliardi, A.; Landi, C.; Carta, S.; Nuti, R.; Giordano, N. Proteomics of human primary osteoarthritic chondrocytes exposed to extremely low-frequency electromagnetic fields (ELF EMFs) and to therapeutic application of musically modulated electromagnetic fields (TAMMEF). Electromagn Biol Med 2014, 33(1), 3-10; doi: 10.3109/15368378.2013.782316
26. Duan, Y.; Wang, Z.; Zhang, H.; He, Y.; Lu, R.; Zhang, R.; Sun, G.; Sun, X. The preventive effect of lotus seedpod procyanidins on cognitive impairment and oxidative damage induced by extremely low frequency electromagnetic field exposure. Food Funct 2013, 4(8), 1252-1262; doi: 10.1039/c3fo60116a
27. Li, S.S.; Zhang, Z.Y.; Yang, C.J.; Lian, H.Y.; Cai, P. Gene expression and reproductive abilities of male Drosophila melanogaster subjected to ELF-EMF exposure. Mutat Res 2013, 758(1-2), 95-103; doi: 10.1016/j.mrgentox.2013.10.004
28. Alsaeed, I.; Al-Somali, F.; Sakhnini, L.; Aljarallah, O.S.; Hamdan, R.M.; Bubishate, S.A.; Sarfaraz, Z.K.; Kamal, A. Autism-relevant social abnormalities in mice exposed perinatally to extremely low frequency electromagnetic fields. Int J Dev Neurosci 2014, 37, 58-64; doi: 10.1016/j.ijdevneu.2014.06.010
29. Giorgi, G.; Lecciso, M.; Capri, M.; Lukas Yani, S.; Virelli, A.; Bersani, F.; Del Re, B. An evaluation of genotoxicity in human neuronal-type cells subjected to oxidative stress under an extremely low frequency pulsed magnetic field. Mutat Res Genet Toxicol Environ Mutagen 2014, 775-776, 31-7; doi: 10.1016/j.mrgentox.2014.10.003

30. de Groot, M.W.; Kock, M.D.; Westerink, R.H. Assessment of the neurotoxic potential of exposure to 50Hz extremely low frequency electromagnetic fields (ELF-EMF) in naive and chemically stressed PC12 cells. Neurotoxicology 2014, 44, 358-364; doi: 10.1016/j.neuro.2014.07.00
31. Komaki, A.; Khalili, A.; Salehi, I.; Shahidi, S.; Sarihi, A. Effects of exposure to an extremely low frequency electromagnetic field on hippocampal long-term potentiation in rat. Brain Res 2014, 1564, 1-8; doi: 10.1016/j.brainres.2014.03.041
32. Li, Y.; Yan, X.; Liu, J.; Li, L.; Hu, X.; Sun, H.; Tian, J. Pulsed electromagnetic field enhances brain-derived neurotrophic factor expression through L-type voltage-gated calcium channel- and Erk-dependent signaling pathways in neonatal rat dorsal root ganglion neurons. Neurochem Int 2014, 75, 96-104; doi: 10.1016/j.neuint.2014.06.004
33. Luo, F.L.; Yang, N.; He, C.; Li, H.L.; Li, C.; Chen, F.; Xiong, J.X.; Hu, Z.A.; Zhang, J. Exposure to extremely low frequency electromagnetic fields alters the calcium dynamics of cultured entorhinal cortex neurons. Environ Res 2014, 135, 236-246; doi: 10.1016/j.envres.2014.09.023
34. Mahdavi, S.M.; Sahraei, H.; Yaghmaei, P.; Tavakoli, H. Effects of electromagnetic radiation exposure on stress-related behaviors and stress hormones in male wistar rats. Biomol Ther (Seoul) 2014, 22(6), 570-576; doi:10.4062/biomolther.2014.054
35. Mattar, F.A.; Bareedy, M.H.; El-Dosouky, M.E.M.; Zaghloul, M.S.; Mahmod, M.S.M. Effects of 6 Weeks Exposure of 3.5 mT (ELF EMF) on Some Animal behaviors in White Albino Rat (Sprague Dawley) Pups, Zagazig Veterinary Journal 2014, 42(1), 74-80
36. Reale, M.; Kamal, M.A.; Patruno, A.; Costantini, E.; D'Angelo, C.; Pesce, M.; Greig, N.H. Neuronal cellular responses to extremely low frequency electromagnetic field exposure: implications regarding oxidative stress and neurodegeneration. PLoS One 2014, 9(8), e104973; doi: 10.1371/journal.pone.0104973
37. Ardeshirylajimi, A.; Soleimani, M. Enhanced growth and osteogenic differentiation of Induced Pluripotent Stem cells by Extremely Low-Frequency Electromagnetic Field. Cell Mol Biol (Noisy-le-grand) 2015, 61(1), 36-41
38. Chung, Y.H.; Lee, Y.J.; Lee, H.S.; Chung, S.J.; Lim, C.H.; Oh, K.W.; Sohn, U.D.; Park, E.S.; Jeong, J.H. Extremely low frequency magnetic field modulates the level of neurotransmitters. Korean J Physiol Pharmacol 2015, 19(1), 15-20; doi: 10.4196/kjpp.2015.19.1.15
39. Duan, W.; Liu, C.; Zhang, L.; He, M.; Xu, S.; Chen, C.; Pi, H.; Gao, P.; Zhang, Y.; Zhong, M.; Yu, Z.; Zhou, Z. Comparison of the genotoxic effects induced by 50 Hz extremely low-frequency electromagnetic fields and 1800 MHz radiofrequency electromagnetic fields in GC-2 cells. Radiat Res 2015, 183(3), 305-314; doi: 10.1667/RR13851.1
40. Golbach, L.A.; Philippi, J.G.; Cuppen, J.J.; Savelkoul, H.F.; Verburg-van Kemenade, B.M. Calcium signalling in human neutrophil cell lines is not affected by low-frequency electromagnetic fields. Bioelectromagnetics 2015, 36(6), 430-43; doi: 10.1002/bem.21924
41. Lewicka, M.; Henrykowska, G.A.; Pacholski, K.; Szczęsny, A.; Dziedziczak-Buczyńska, M.; Buczyński, A. The impact of electromagnetic radiation of different parameters on platelet oxygen metabolism - in vitro studies. Adv Clin Exp Med 2015, 24(1), 31-35; doi: 10.17219/acem/38169
42. Li, L.; Xiong, D.F.; Liu, J.W.; Li, Z.X.; Zeng, G.C.; Li, H.L. A cross-sectional study on oxidative stress in workers exposed to extremely low frequency electromagnetic fields. Int J Radiat Biol 2015, 91(5), 420-425; doi: 10.3109/09553002.2015.1012304. PMID: 25651040
43. Liu, X.; Zuo, H.; Wang, D.; Peng, R.; Song, T.; Wang, S.; Xu, X.; Gao, Y.; Li, Y.; Wang, S.; Wang, L.; Zhao, L. Improvement of spatial memory disorder and hippocampal damage by exposure to electromagnetic fields in an Alzheimer's disease rat model. PLoS One 2015, 10(5):e0126963; doi: 10.1371/journal.pone.0126963
44. Patruno, A.; Tabrez, S.; Pesce, M.; Shakil, S.; Kamal, M.A.; Reale, M. Effects of extremely low frequency electromagnetic field (ELF-EMF) on catalase, cytochrome P450 and nitric oxide synthase in erythro-leukemic cells. Life Sci 2015, 121, 117-123; doi: 10.1016/j.lfs.2014.12.003
45. Tiwari, R.; Lakshmi, N.K.; Bhargava, S.C.; Ahuja, Y.R. Epinephrine, DNA integrity and oxidative stress in workers exposed to extremely low-frequency electromagnetic fields (ELF-EMFs) at 132 kV substations. Electromagn Biol Med 2015, 34(1), 56-62; doi: 10.3109/15368378.2013.869755

46. Yang, M.L.; Ye, Z.M. [Extremely low frequency electromagnetic field induces apoptosis of osteosarcoma cells via oxidative stress]. Zhejiang Da Xue Xue Bao Yi Xue Ban 2015, 44(3), 323-328. Chinese
47. Zhao, Q.R.; Lu, J.M.; Yao, J.J.; Zhang, Z.Y.; Ling, C.; Mei, Y.A. Neuritin reverses deficits in murine novel object associative recognition memory caused by exposure to extremely low-frequency (50 Hz) electromagnetic fields. Sci Rep 2015, 5, 11768; doi: 10.1038/srep11768
48. Falone, S.; Marchesi, N.; Osera, C.; Fassina, L.; Comincini, S.; Amadio, M.; Pascale, A. Pulsed electromagnetic field (PEMF) prevents pro-oxidant effects of H2O2 in SK-N-BE(2) human neuroblastoma cells. Int J Radiat Biol 2016, 92(5), 281-286; doi: 10.3109/09553002.2016.1150619
49. Ferroni, L.; Tocco, I.; De Pieri, A.; Menarin, M.; Fermi, E.; Piattelli, A.; Gardin, C.; Zavan, B. Pulsed magnetic therapy increases osteogenic differentiation of mesenchymal stem cells only if they are pre-committed. Life Sci 2016, 152, 44-51; doi: 10.1016/j.lfs.2016.03.020
50. de Kleijn, S.; Ferwerda, G.; Wiese, M.; Trentelman, J.; Cuppen, J.; Kozicz, T.; de Jager, L.; Hermans, P.W.; Verburg-van Kemenade, B.M. A short-term extremely low frequency electromagnetic field exposure increases circulating leukocyte numbers and affects HPA-axis signaling in mice. Bioelectromagnetics 2016, 37(7), 433-443; doi: 10.1002/bem.21998
51. Lai, J.; Zhang, Y.; Liu, X.; Zhang, J.; Ruan, G.; Chaugai, S.; Chen, C.; Wang, D.W. Effects of extremely low frequency electromagnetic fields (100μT) on behaviors in rats. Neurotoxicology 2016, 52, 104-113; doi: 10.1016/j.neuro.2015.11.010
52. Luo, X.; Chen, M.; Duan, Y.; Duan, W.; Zhang, H.; He, Y.; Yin, C.; Sun, G.; Sun, X. Chemoprotective action of lotus seedpod procyanidins on oxidative stress in mice induced by extremely low-frequency electromagnetic field exposure. Biomed Pharmacother 2016, 82, 640-648; doi: 10.1016/j.biopha.2016.06.005
53. Nakayama, M.; Nakamura, A.; Hondou, T.; Miyata, H. Evaluation of cell viability, DNA single-strand breaks, and nitric oxide production in LPS-stimulated macrophage RAW264 exposed to a 50-Hz magnetic field. Int J Radiat Biol 2016, 92(10), 583-589; doi: 10.1080/09553002.2016.1206224
54. Sun, Z.C.; Ge, J.L.; Guo, B.; Guo, J.; Hao, M.; Wu, Y.C.; Lin, Y.A.; La, T.; Yao, P.T.; Mei, Y.A.; Feng, Y.; Xue, L. Extremely Low Frequency Electromagnetic Fields Facilitate Vesicle Endocytosis by Increasing Presynaptic Calcium Channel Expression at a Central Synapse. Sci Rep 2016, 6, 21774; doi: 10.1038/srep21774
55. Wei, J.; Tong, J.; Yu, L.; Zhang, J. EMF protects cardiomyocytes against hypoxia-induced injury via heat shock protein 70 activation. Chem Biol Interact 2016, 248, 8-17; doi: 10.1016/j.cbi.2016.02.003
56. Wyszkowska, J.; Shepherd, S.; Sharkh, S.; Jackson, C.W.; Newland, P.L. Exposure to extremely low frequency electromagnetic fields alters the behaviour, physiology and stress protein levels of desert locusts. Sci Rep 2016, 6, 36413; doi: 10.1038/srep36413
57. Yin, C.; Luo, X.; Duan, Y.; Duan, W.; Zhang, H.; He, Y.; Sun, G.; Sun, X. Neuroprotective effects of lotus seedpod procyanidins on extremely low frequency electromagnetic field-induced neurotoxicity in primary cultured hippocampal neurons. Biomed Pharmacother 2016 , 82, 628-639; doi: 10.1016/j.biopha.2016.05.032
58. Zhang, Y.; Zhang, D.; Zhu, B.; Zhang, H.; Sun, Y.; Sun, C. Effects of dietary green tea polyphenol supplementation on the health of workers exposed to high-voltage power lines. Environ Toxicol Pharmacol 2016, 46, 183-187; doi: 10.1016/j.etap.2016.07.016
59. Zhu, K.; Lv, Y.; Cheng, Q.; Hua, J.; Zeng, Q. Extremely Low Frequency Magnetic Fields Do Not Induce DNA Damage in Human Lens Epithelial Cells In Vitro. Anat Rec (Hoboken) 2016, 299(5), 688-697; doi: 10.1002/ar.23312
60. Calcabrini, C.; Mancini, U.; De Bellis, R.; Diaz, A.R.; Martinelli, M.; Cucchiarini, L.; Sestili, P.; Stocchi, V.; Potenza, L. Effect of extremely low-frequency electromagnetic fields on antioxidant activity in the human keratinocyte cell line NCTC 2544. Biotechnol Appl Biochem 2017, 64(3), 415-422; doi: 10.1002/bab.1495

61. Cichoń, N.; Bijak, M.; Miller, E.; Saluk, J. Extremely low frequency electromagnetic field (ELF-EMF) reduces oxidative stress and improves functional and psychological status in ischemic stroke patients. Bioelectromagnetics 2017, 38(5), 386-396; doi: 10.1002/bem.22055
62. Djordjevic, N.Z.; Paunović, M.G.; Peulić, A.S. Anxiety-like behavioural effects of extremely low-frequency electromagnetic field in rats. Environ Sci Pollut Res Int 2017, 24(27), 21693-21699; doi: 10.1007/s11356-017-9710-1
63. Ehnert, S.; Fentz, A. K.; Schreiner, A.; Birk, J.; Wilbrand, B.; Ziegler, P.; Reumann, M. K.; Wang, H.; Falldorf, K.; Nussler, A. K. Extremely low frequency pulsed electromagnetic fields cause antioxidative defense mechanisms in human osteoblasts via induction of •O2- and H2O2. Scientific reports 2017, 7(1), 14544; doi.org/10.1038/s41598-017-14983-9
64. Haghighat, N.; Abdolmaleki, P.; Parnian, J.; Behmanesh, M. The expression of pluripotency and neuronal differentiation markers under the influence of electromagnetic field and nitric oxide. Mol Cell Neurosci 2017, 85, 19-28; doi: 10.1016/j.mcn.2017.08.005
65. Kuzay, D.; Ozer, C.; Sirav, B.; Canseven, A.G.; Seyhan, N. Oxidative effects of extremely low frequency magnetic field and radio frequency radiation on testes tissues of diabetic and healthy rats. Bratisl Lek Listy 2017, 118(5), 278-282; doi: 10.4149/BLL_2017_055
66. Sakhaie, M.H.; Soleimani, M.; Pourheydar, B.; Majd, Z.; Atefimanesh, P.; Asl, S.S.; Mehdizadeh, M. Effects of Extremely Low-Frequency Electromagnetic Fields on Neurogenesis and Cognitive Behavior in an Experimental Model of Hippocampal Injury. Behav Neurol 2017, 2017, 9194261; doi: 10.1155/2017/9194261
67. Urnukhsaikhan, E.; Mishig-Ochir, T.; Kim, S.C.; Park, J.K.; Seo, Y.K. Neuroprotective Effect of Low Frequency-Pulsed Electromagnetic Fields in Ischemic Stroke. Appl Biochem Biotechnol 2017, 181(4), 1360-1371; doi: 10.1007/s12010-016-2289-z
68. Budziosz, J.; Stanek, A.; Sieroń, A.; Witkoś, J.; Cholewka, A.; Sieroń, K. Effects of Low-Frequency Electromagnetic Field on Oxidative Stress in Selected Structures of the Central Nervous System. Oxid Med Cell Longev 2018, 2018:1427412; doi: 10.1155/2018/1427412
69. Burman, O.; Marsella, G.; Di Clemente, A.; Cervo, L. The effect of exposure to low frequency electromagnetic fields (EMF) as an integral part of the housing system on anxiety-related behaviour, cognition and welfare in two strains of laboratory mouse. PLoS One 2018, 13(5):e0197054; doi: 10.1371/journal.pone.0197054
70. Cichoń, N.; Rzeźnicka, P.; Bijak, M.; Miller, E.; Miller, S.; Saluk, J. Extremely low frequency electromagnetic field reduces oxidative stress during the rehabilitation of post-acute stroke patients. Adv Clin Exp Med 2018A, 27(9), 1285-1293; doi: 10.17219/acem/73699
71. Cichoń, N.; Bijak, M.; Czarny, P.; Miller, E.; Synowiec, E.; Sliwinski, T.; Saluk-Bijak, J. Increase in Blood Levels of Growth Factors Involved in the Neuroplasticity Process by Using an Extremely Low Frequency Electromagnetic Field in Post-stroke Patients. Front Aging Neurosci 2018B, 10, 294; doi: 10.3389/fnagi.2018.00294
72. Fathi, E.; Farahzadi, R. Zinc Sulphate Mediates the Stimulation of Cell Proliferation of Rat Adipose Tissue-Derived Mesenchymal Stem Cells Under High Intensity of EMF Exposure. Biol Trace Elem Res 2018, 184(2), 529-535; doi: 10.1007/s12011-017-1199-4
73. Laszlo, A.M.; Ladanyi, M.; Boda, K.; Csicsman, J.; Bari, F.; Serester, A.; Molnar, Z.; Sepp, K.; Galfi, M.; Radacs, M. Effects of extremely low frequency electromagnetic fields on turkeys. Poult Sci 2018, 97(2), 634-642; doi: 10.3382/ps/pex304
74. Martínez-Sámano, J.; Flores-Poblano, A.; Verdugo-Díaz, L.; Juárez-Oropeza, M.A.; Torres-Durán, P.V. Extremely low frequency electromagnetic field exposure and restraint stress induce changes on the brain lipid profile of Wistar rats. BMC Neurosci 2018, 19(1), 31; doi: 10.1186/s12868-018-0432-1
75. Rezaei-Tavirani, M.; Hasanzadeh, H.; Seyyedi, S.; Ghoujeghi, F.; Semnani, V.; Zali, H. Proteomic Analysis of Extremely Low-Frequency ElectroMagnetic Field (ELF-EMF) With Different Intensities in Rats Hippocampus, Arch Neurosci 2018, 5(1):e62954; doi: 10.5812/archneurosci.62954

76. Song, K.; Im, S.H.; Yoon, Y.J.; Kim, H.M.; Lee, H.J.; Park, G.S. A 60 Hz uniform electromagnetic field promotes human cell proliferation by decreasing intracellular reactive oxygen species levels. PLoS One 2018, 13(7), e0199753; doi:10.1371/journal.pone.0199753
77. Sun, Y.; Shi, Z.; Wang, Y.; Tang, C.; Liao, Y.; Yang, C.; Cai, P. Coupling of oxidative stress responses to tricarboxylic acid cycle and prostaglandin E2 alterations in Caenorhabditis elegans under extremely low-frequency electromagnetic field. Int J Radiat Biol 2018, 94(12), 1159-1166; doi: 10.1080/09553002.2019.1524943
78. Wyszkowska, J.; Jędrzejewski, T.; Piotrowski, J.; Wojciechowska, A.; Stankiewicz, M.; Kozak W. Evaluation of the influence of in vivo exposure to extremely low-frequency magnetic fields on the plasma levels of pro-inflammatory cytokines in rats. Int J Radiat Biol 2018, 94(10), 909-917; doi: 10.1080/09553002.2018.1503428
79. Cichoń, N.; Saluk-Bijak, J.; Miller, E.; Sliwinski, T.; Synowiec, E.; Wigner, P.; Bijak, M. Evaluation of the effects of extremely low frequency electromagnetic field on the levels of some inflammatory cytokines in post-stroke patients. J Rehabil Med 2019, 51(11), 854-860; doi: 10.2340/16501977-262
80. Hosseinabadi, M.B.; Khanjani, N. The Effect of Extremely Low-Frequency Electromagnetic Fields on the Prevalence of Musculoskeletal Disorders and the Role of Oxidative Stress. Bioelectromagnetics 2019A, 40(5), 354-360; doi: 10.1002/bem.22198
81. Hosseinabadi, M.B.; Khanjani, N.; Ebrahimi, M.H.; Haji, B.; Abdolahfard, M. The effect of chronic exposure to extremely low-frequency electromagnetic fields on sleep quality, stress, depression and anxiety. Electromagn Biol Med 2019B, 38(1), 96-101; doi: 10.1080/15368378.2018.1545665
82. Karimi, S.A.; Salehi, I.; Shykhi, T.; Zare, S.; Komaki, A. Effects of exposure to extremely low-frequency electromagnetic fields on spatial and passive avoidance learning and memory, anxiety-like behavior and oxidative stress in male rats. Behav Brain Res 2019, 359, 630-638; doi: 10.1016/j.bbr.2018.10.002
83. Merla, C.; Liberti, M.; Consales, C.; Denzi, A.; Apollonio, F.; Marino, C.; Benassi, B. Evidences of plasma membrane-mediated ROS generation upon ELF exposure in neuroblastoma cells supported by a computational multiscale approach. Biochim Biophys Acta Biomembr 2019, 1861(8), 1446-1457; doi: 10.1016/j.bbamem.2019.06.005
84. Sun, Y.; Huang, X.; Wang, Y.; Shi, Z.; Liao, Y.; Cai, P. Lipidomic alteration and stress-defense mechanism of soil nematode Caenorhabditis elegans in response to extremely low-frequency electromagnetic field exposure. Ecotoxicol Environ Saf 2019, 170, 611-619; doi: 10.1016/j.ecoenv.2018.11.137
85. Mahaki, H.; Jabarivasal, N.; Sardanian, K.; Zamani, A. Effects of Various Densities of 50 Hz Electromagnetic Field on Serum IL-9, IL-10, and TNF-α Levels. Int J Occup Environ Med 2020, 11(1), 24-32; doi: 10.15171/ijoem.2020.1572
86. Touitou, Y.; Lambrozo, J.; Mauvieux, B.; Riedel, M. Evaluation in humans of ELF-EMF exposure on chromogranin A, a marker of neuroendocrine tumors and stress. Chronobiol Int 2020, 37(1), 60-67; doi: 10.1080/07420528.2019.1683857
